# Cockayne syndrome B protein is implicated in transcription and associated chromatin dynamics in homeostatic and genotoxic conditions

**DOI:** 10.1111/acel.14341

**Published:** 2024-10-06

**Authors:** Anastasios Liakos, Katerina Z. Ntakou‐Zamplara, Nelina Angelova, Dimitris Konstantopoulos, Anna‐Chloe Synacheri, Zoi Spyropoulou, Iason A. Tsarmaklis, Despoina Korrou‐Karava, Georgios Nikolopoulos, Matthieu D. Lavigne, Maria Fousteri

**Affiliations:** ^1^ Institute for Fundamental Biomedical Research BSRC “Alexander Fleming” Vari Greece; ^2^ Laboratory of Biology, School of Medicine National and Kapodistrian University of Athens (NKUA) Athens Greece; ^3^ Present address: Department of Physiology, School of Medicine University of Patras Patras Greece; ^4^ Present address: Institute of Molecular Biology & Biotechnology, FORTH Crete Greece

**Keywords:** chromatin dynamics, Cockayne syndrome, DNA damage response, genome integrity mechanisms, transcription regulation

## Abstract

The integrity of the actively transcribed genome against helix‐distorting DNA lesions relies on a multilayered cellular response that enhances Transcription‐Coupled Nucleotide Excision Repair (TC‐NER). When defective, TC‐NER is causatively associated with Cockayne‐Syndrome (CS), a rare severe human progeroid disorder. Although the presence of unresolved transcription‐blocking lesions is considered a driver of the aging process, the molecular features of the transcription‐driven response to genotoxic stress in CS‐B cells remain largely unknown. Here, an in‐depth view of the transcriptional and associated chromatin dynamics that occur in CS‐B cells illuminates the role of CSB therein. By employing high‐throughput genome‐wide approaches, we observed that absence of a functional CSB protein results in a delay in transcription progression, more positioned +1 nucleosomes, and less dynamic chromatin structure, compared to normal cells. We found that early after exposure to UV, CS‐B cells released RNA polymerase II (RNAPII) from promoter‐proximal pause sites into elongation. However, the magnitude of this response and the progression of RNAPII were reduced compared to normal counterparts. Notably, we detected increased post‐UV retainment of unprocessed nascent RNA transcripts and chromatin‐associated elongating RNAPII molecules. Contrary to the prevailing models, we found that transcription initiation is operational in CS‐B fibroblasts early after UV and that chromatin accessibility showed a marginal increase. Our study provides robust evidence for the role of CSB in shaping the transcription and chromatin landscape both in homeostasis and in response to genotoxic insults, which is independent of its known role in TC‐NER, and which may underlie major aspects of the CS phenotype.

## Graphical abstract

Early after exposure to UV, RNAPII is released from promoter‐proximal pause sites to elongation in both normal and CS‐B fibroblasts. However, RNAPII progression speed is slower in CS‐B compared to normal fibroblasts. Additionally, UV‐exposed CS‐B cells are characterized by increased retention of nascent RNA transcripts and elongating RNAPII molecules in gene bodies, minimal changes in chromatin accessibility and, more compact chromatin structure, compared to irradiated normal cells.

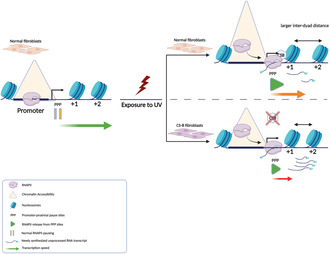

**Abbreviations**
ATAC‐seq: assay for transposase‐accessible chromatin with sequencing
ChIP: chromatin immunoprecipitation
CPD: cyclobutane Pyrimidine Dimers
CS: Cockayne‐Syndrome
DMEM: Dulbecco's modified eagle medium
DRB: 5,6‐dichloro‐1‐β‐D‐ribofuranosylbenzimidazole
EI: Escape index
EU: 5‐ethynyl uridine
GG‐NER: global‐genome nucleotide excision repair
GRO‐seq: global run‐on‐sequencing
HMM: Hidden Markov Model
NGS: next generation sequencing
nRNA: nascent RNA
PBS: phosphate‐buffered saline
PPP: promoter proximal pause sites
PVDF: polyvinylidene fluoride membrane
qPCR: quantitative polymerase chain reaction
RNAPII: RNA polymerase II
RPKM: reads per kilobase of transcript, per million mapped reads
SEM: standard error of the mean
TCA: trichloroacetic acid
TC‐NER: transcription‐coupled nucleotide excision repair
TSS: transcription start site
TTS: transcription termination site
UV: ultraviolet irradiation
XR‐seq: excision repair sequencing

## 1 INTRODUCTION

All organisms must maintain their genome integrity in the face of environmental and endogenous DNA damage factors. Several DNA repair mechanisms have been developed with great specificity depending on the type of lesion (Chatterjee & Walker, 2017). Nucleotide excision repair (NER), a highly conserved mechanism, can recognize and remove helix‐distorting DNA adducts such as the ones induced by Ultraviolet irradiation (UV)‐, cigarette smoke‐ and cisplatin via the action of its two sub‐pathways. Specifically, Transcription Coupled Nucleotide Excision Repair (TC‐NER) subpathway is coupled to active transcription and can efficiently remove lesions that block the progression of RNA polymerase II (RNAPII) whereas Global Genome Nucleotide Excision Repair (GG‐NER) subpathway is responsible for removing lesions from the entire genome (Marteijn et al., 2014; Spivak, 2015).

One of the first steps of the TC‐NER cascade is the sequential recruitment of Cockayne Syndrome (CS) CSB and CSA proteins on the DNA template, in transcribed genomic regions of active genes where RNA Polymerase II (RNAPII) stalls at bulky DNA lesions (Vermeulen & Fousteri, 2013). Their functional importance becomes evident as mutations in either *ERCC6* (encodes for CSB) or *ERCC8* (encodes for CSA) genes give rise to CS in humans. Patients with this clinically heterogeneous and multisystemic progeroid disorder present a range of developmental abnormalities and aging‐associated pathologies including progressive neurodegeneration, mental retardation, microcephaly, growth failure, severe neurological dysfunction, atherosclerosis, osteoporosis, retinal degeneration, and liver and kidney aging before the age of 10 (Karikkineth et al., 2017; Laugel, 2013; Liakos et al., 2017; Schumacher et al., 2021).

Apart from triggering TC‐NER, exposure to UV exerts a broad‐ranging impact on various transcriptional and co‐transcriptional processes (Heilbrun et al., 2020; Nieto Moreno et al., 2023), operating at multiple levels of the cellular stress response. It has been shown that in normal fibroblasts, a dynamic and synchronous global release of RNAPII molecules from promoter‐proximal pause (PPP) sites takes place in response to UV‐irradiation (Borisova et al., 2018; Lavigne et al., 2017; Liakos et al., 2020). This mechanism leads to increased damage sensing and DNA repair and ensures uniform and accelerated surveillance for the whole transcribed genome (Lavigne et al., 2017; Liakos et al., 2020). This process is fed by increased recruitment of RNAPII at the transcription start sites (TSSs) (Liakos et al., 2023) and elevated nascent (n) RNA synthesis at the 5′ ends of active genes (Andrade‐Lima et al., 2015; Lavigne et al., 2017; Liakos et al., 2020; Magnuson et al., 2015; Williamson et al., 2017).

Concerning CS‐B fibroblasts, in addition to their inability to restore RNA synthesis in bulk after exposure to UV radiation (Mayne & Lehmann, 1982), early studies suggested that the process of transcription initiation remains permanently inhibited after induction of DNA damage (Proietti‐De‐Santis et al., 2006; Rockx et al., 2000). While it has been proposed that prolonged and not resolved action of transcription repressors control this process (Epanchintsev et al., 2017; Kristensen et al., 2013), another study by Andrade Lima et al., (Andrade‐Lima et al., 2015) reported an increase of nascent transcription at the 5′ end of genes in CS‐B fibroblasts for up to 6 h after UV irradiation. In the latter study, the authors pointed to a lack of transcription recovery at the 3′ end of genes, suggesting that the primary cause of aberration in the transcriptional UV response of CS‐B cells lies in defects related to the progression of RNAPII elongation, rather than transcription initiation. Thus, although several molecular aspects of the UV response in normal fibroblasts have been identified over the past years, the exact mechanisms through which these responses are mediated in CS‐B fibroblasts remain unclear.

In this study, we undertook a systematic approach to uncover the molecular aspects of the cellular response to UV in CS‐B fibroblasts and shed light on CSB protein's role in steady state and upon genotoxic stress. Our results revealed that under both control and irradiated conditions, CS‐B cells exhibited a delay in transcription progression through the genes, in line with a more compact and less dynamic chromatin structure at genomic regions close to TSSs, compared to normal fibroblasts. We found that early after damage induction, RNAPII molecules are rapidly released from PPP sites into productive elongation to create a wave of nascent transcription in CS‐B cells similar to UV‐exposed TC‐NER‐proficient normal fibroblasts. Nevertheless, this transcription‐driven response resulted in prolonged retention of incomplete nascent transcripts and chromatin‐bound elongating RNAPII molecules compared to normal cells. Remarkably, we demonstrated that up to 4 h after exposure to UV irradiation transcription initiation continues to operate in CS‐B fibroblasts while no major changes were observed in chromatin accessibility and H3K27ac signal at regulatory regions. We also report an increased association of CSB with elongating RNAPII at the 5′ of genes in normal human fibroblasts, which is further potentiated and shifted towards the gene bodies upon UV exposure, suggestive for a role of CSB protein in the UV‐triggered RNAPII progression in the gene bodies of active genes. Collectively, this work provides novel insights into the significant roles of CSB protein in steady state and in the multilayered response to genotoxic insult, uncoupling it from its established role in TC‐NER, and providing a different perspective on the causative links between transcriptional dysfunction and the etiology of CS phenotype.

## 2 METHODS

### 2.1 Cell culture

Cell lines used in this study were normal human skin fibroblasts (VH10) htert immortalised (Overmeer et al., 2010; Tresini et al., 2015), CS1AN human skin fibroblasts (CS‐B) htert immortalised (Vrouwe et al., 2011), and CS1AN human fibroblasts SV40 immortalized expressing (or not) the wild type CSB protein tagged with an HA epitope (HA CS‐B‐[His]_6_) (Van Gool et al., 1997). Cells were grown under standard culture conditions in Dulbecco's Modified Eagle Medium (DMEM, Thermo Scientific) supplemented with 10% v/v fetal bovine serum (FBS, Thermo Scientific) and 1% v/v penicillin–streptomycin (Thermo Scientific), in a humidified incubator at 37°C and 5% CO_2_. Confluent cells were synchronized at the G_0_/G_1_ phase by serum deprivation for 72 h (DMEM with 0.5% FBS) followed by the release in full serum medium (10%) for 3 h before treatment appropriate for each condition.

The cells were exposed to UV‐C radiation (254 nm, TUV Lamp, Philips) at 8 J/m^2^–20 J/m^2^ doses (indicated in each experiment), then left to recover in a fully supplemented medium. Transcription inhibitor 5, 6‐Dichloro‐1‐β‐D‐ribofuranosylbenzimidazole (DRB, Calbiochem) was applied directly to the growth medium and was used at a final concentration of 100 μM, at the indicated time points.

### 2.2 ChIP, ChIP‐qPCR, ChIP‐seq

ChIP was performed as previously described (Lavigne et al., 2017; Liakos et al., 2020). Briefly, 100–150 μg of sonicated crosslinked chromatin were used for each experimental condition and incubated with the indicated antibody. The following antibodies were used: anti‐RNAPII‐Ser2P (Abcam, ab5095, 4 μg per ChIP), anti‐RNAPII‐hypo (Millipore, 05‐952‐I‐25, 2.5 μg per ChIP), anti‐CSB (Santa Cruz, sc 104–59, 5 μg per ChIP), anti‐H3K27ac (Abcam, ab4729, 2.5 μg per ChIP). The immunoprecipitated DNA was quantified using the Qubit 2.0 Fluorometer system (dsDNA HS Assay Kit, Thermo Scientific) and validated through ChIP‐qPCR. For ChIP‐qPCR experiments 30–100 pg of ChIP and Input DNA were used in duplicate reactions using qPCRBIO SyGreen mix (PCR BIOSYSTEMS, Cat No PB20.14–50) in a Roche Light Cycler 96 instrument. The primers used are listed in Table S1. The quality of ChIP‐seq libraries was assayed using Agilent High Sensitivity Bioanalyzer and NGS sequencing was performed at Genecore Genomics Core Facility (EMBL, Heidelberg).

### 2.3 Acetic extraction of histone proteins

Histone extracts were prepared as previously described (Liakos et al., 2020). Briefly, confluent CS1AN htert cells were synchronized by low serum and released in a normal medium as described above. Irradiated cells were treated with 15 J/m^2^. Cells were harvested, washed 3 times with ice‐cold PBS, lysed in 10 volumes of Lysis Buffer (10 mM Hepes pH 7.9, 1.5 mM MgCl_2_, 10 mM KCl, 0.5 mM DTT, 1.5 mM PMSF, 0.2 M sulfuric acid) and kept on ice for 30 min. Next, the suspension was centrifuged at 10,080 rcf for 10 min at 4°C, supernatant was collected and TCA was added to a final concentration of 20%. After mild vortexing, samples were incubated on ice for 1 h and centrifuged for 15 min at 18,600 rcf at 4°C. The supernatant was discarded and the pellet was washed with ice‐cold acetone. After centrifugation, for 5 min at 18,600 rcf at 4°C, acetone was removed, the pellet was air‐dried and resuspended in TE buffer. Before acrylamide gel electrophoresis, an appropriate volume of Laemmli Buffer was added and histone extracts were boiled for 15 min.

### 2.4 Western blot analysis

Western blot analysis was performed using crosslinked chromatin extracts on custom‐made polyacrylamide gels (polyacrylamide concentration 6%–16%) or pre‐cast gradient gels (gradient gel, NuPAGE 4%–12% Bis‐Tris Protein Gels, Thermo Fisher). The proteins were then transferred to a Polyvinylidene Fluoride (PVDF) membrane (Merck, cat. no. IPFL00010), blocked with Odyssey Blocking Buffer (Li‐Cor, cat. no. 927–40,000) at 4°C overnight, and stained with the following antibodies: anti‐RNAPII‐Ser2P (ab5095, Abcam, 1:1000 dilution), anti‐RNAPII‐hypo (8WG16, 05‐952‐I‐25, Millipore, 1:500 dilution), anti‐H3K27ac (ab4729, Abcam, 1:1000), anti‐H2B (07–371, Millipore, 1:1000), anti‐H4 (ab10158, Abcam, 1:1000). Then the membrane was incubated with the appropriate secondary antibodies (dilution 1:10.000), for 1 h at room temperature, visualized with the Odyssey CLx system (Li‐Cor) and analyzed with the Image Studio software (Li‐Cor). The antibodies were diluted according to the instructions of their respective manufacturer.

### 2.5 Assay for transposase‐accessible chromatin (ATAC)‐seq

ATAC‐seq was performed using the Nextera DNA Library Prep kit (Illumina, Inc.) as previously described (Liakos et al., 2020) and based on the omni‐ATAC‐seq protocol (Corces et al., 2017). 100,000 cells were used for each experimental condition. Cells were either mock‐treated or UV‐irradiated with a dose of 15 J/m^2^ and then allowed to recover for the indicated time points before harvesting. Size distribution and quality of ATAC‐seq libraries were checked through High Sensitivity Agilent Bioanalyzer. Paired‐end sequencing of ATAC libraries was performed at Genecore (EMBL, Heidelberg) using the NextSeq 500 Illumina platform.

### 2.6 GRO‐seq

For DRB GRO‐seq experiments, confluent fibroblasts were treated with DRB (100 μM) for 3 h, allowing clearance of transcribing gene bodies from elongating RNAPII. nRNA was labeled through the addition of 5‐ethynyl uridine (EU, 0.1 mM) for 10 min, at the time points depicted. nRNA was isolated with Trizol, captured using Click‐iT Nascent RNA (nRNA) Capture kit (Thermo Fisher Scientific, Cat. Number C10365), biotinylated using 0.5 mM biotin azide and bound to streptavidin beads. nRNA fragmentation, first and second‐strand synthesis were performed as previously described (Lavigne et al., 2017).

### 2.7 Pulse Chase‐seq

For the Pulse Chase analysis, nRNA was labeled with the addition of 5‐ethynyl uridine (EU, 0.1 mM) for 1 h, 30 min before UV irradiation and 30 min after, then EU was removed and the cells were left to recover before total RNA extraction with TRIzol (Invitrogen, #15596026) at the time points specified. After DNAse I treatment, EU‐labeled molecules were isolated using the Click‐iT™ kit (nRNA Capture Kit, C10365, ThermoFisher Scientific) according to the manufacturer's protocol. rRNA was depleted using the Ribo‐Zero Magnetic kit (Epicentre, #MRZH11124) as per the manufacturer's instructions. For cDNA synthesis, the SuperScript™ II Reverse Transcriptase Kit (Cat # 18064014) was used. For cDNA synthesis of nRNA, a slightly modified protocol was employed whereby nRNA was fragmented and synthesis was performed on streptavidin beads. Eluted DNA was purified with AMPure XP beads and measured on Qubit 2.0 before library preparation for Next Generation Sequencing (NGS).

### 2.8 Genome annotation

The TSS annotation was based on all known protein‐coding and non‐coding RNA RefSeq transcripts release 86, which were retrieved from the UCSC table browser, using the hg19 genome build (http://genome‐euro.ucsc.edu/cgi‐bin/hgTables). For any Refseq gene model that contained more than one transcript, all elements were clustered together using a 50 bp TSS radius and the longest transcript was selected, resulting in 30.473 TSSs. Of those, only protein‐coding and lncRNA genes were kept, after using the BioMart database (www.biomart.org) for biotype classification, referred to as “mRNAs” in this study, and only mRNAs > = 2 Kb were kept for further analysis, resulting in 25.778 genes. The gene annotations used for the escape index (EI) and nucleosome positioning analyses, underwent an extra filtering step, where overlapping TSSs (within a distance of −250 bp to +2 Kb from a neighboring TSS) were excluded to obtain the genes with the clearest signal, in each case.

### 2.9 Activity determination of annotated mRNAs

To study the process of transcription reorganization using the aforementioned mRNA set as a reference in CS‐B cells, transcripts were classified into three categories based on their transcriptional profile. Each element's TSS was extended from −250 bp to +2 Kb, and the extended genomic elements were intersected with RNAPII Ser2P NO UV, H3K27ac NO UV, and H3K27me3 NO UV peak sets. Regions that overlapped with RNAPII Ser2P and H3K27ac peaks, were categorized as active. Regions overlapping with H3K27me3 peaks, but not with RNAPII Ser2P and H3K27ac peaks were categorized as repressed. Finally, regions with no overlap with any of the aforementioned peak sets were categorized as inactive. Elements that overlapped with both H3K27ac and H3K27me3 peaks were considered as ambiguous, and were excluded from the annotation. The activity categorization resulted in 13,641 active, 1741 repressed and 10,133 inactive TSSs in CS‐B cells (Table S2). For the VH10 cells, only active lists were produced with the data from Liakos et al. (14,229 active genes, 7936 for EI calculations after excluding overlapping TSSs, Table S3). For HA CS‐B cells, an active set for EIs was determined using the RNAPII NOUV peak signals in TSSs (7514 active genes, Table S4).

### 2.10 TSS/intergenic/intragenic/TTS assignment of peaks

The peak sets generated for each condition out of the ATAC‐seq data were pooled together and merged with *bedtools merge* (v2.30.0, (Quinlan & Hall, 2010)). These regions were assigned to position annotations emerging from the RefSeq list of genes: genes smaller than 2 Kb were excluded. The remaining positions were split to: TSS regions (+/− 0.5 Kb from TSS point), Intragenic regions (TSS + 500 bp to TTS 500 bp), TTSs regions (+/− 0.5 Kb from TTS point) and Intergenic regions, which represent the remaining loci. The pooled peaks were intersected with each of the categories and divided to TSS (18.366), Intragenic (154,820), TTSs (4930) and Intergenic (99,989) peaks. The signal of the ATAC‐seq experiment of each condition was projected into these categories to highlight any differences among chromatin accessibility across categories and treatments. Plotting was performed using the *deeptools* package (v3.5.1, (Ramírez et al., 2016)). To assess the increasing or decreasing accessibility by directly comparing conditions, read density heatmaps of UV/+UV ATAC peaks were created through counting the depth of each peak called region with *bam2bed* (*bedops*, v2.4.39, (Neph et al., 2012)) and then *coverageBed —counts*, as in the EI analyses.

### 2.11 Pre‐processing of FASTQ files

Adapter clipping and quality trimming was performed with *cutadapt* (v1.18, (Martin, 2011)). Mapping was performed using *bwa‐mem* (v0.7.17, on https://arxiv.org/abs/1303.3997v2), with default parameters and *hg19* as the reference human genome. HISAT2 (v2.2.1, (Kim et al., 2019)) was used specifically for the Pulse Chase analysis. Hits with a low MAPQ score were filtered out using *samtools* (v1.9, (Danecek et al., 2021)). Chimeric and secondary alignments were also filtered out, and alignments were allowed at most two mismatches between subject and reference sequences, to account for sequencing errors and SNPs between the reference cell line and the sequenced genome. In the case of paired‐end reads, only proper paired‐mates are kept and deduplication was applied using *samtools' markdup*. Blacklisted regions were also excluded and only unique reads were kept when needed depending on the dataset and its purposes. If replicates were present in the dataset, they were concatenated using samtools merge, after being downsampled for library size normalization, and checked for their correlation and profile resemblance. Library size normalization was also corrected among conditions. BigWig files were generated with *deeptools' bamCoverage* and an RPKM normalization.

### 2.12 Peak calling

To identify genomic regions significantly enriched with ATAC‐seq or ChIP‐seq signal, peak calling is applied. For all subsequent peak calling methods mentioned, peak calling is performed at the merged datasets, if replicates are available, except for the Genrich algorithm where replicates are combined internally. Different peak‐calling approaches were used, based on the type of the experiment involved and its scope. CSB ChIP‐seq datasets were analyzed using MACS2 (v2.2.7.1, (Zhang et al., 2008)) in its “narrow” mode. ChIP‐seq peak calling was applied using a control library to model the background signal. For histone modifications ChIP‐seq data, peak calling was performed using epic2 (v0.0.41, (Stovner & Sætrom, 2019)) due to the broader nature of the signal in such data. The epic2 parameters used for both H3K27me3 and H3K27ac datasets were —bin‐size 400 —gaps‐allowed 1 —false‐discovery‐rate‐cutoff 0.01. The same was applied to the RNAPII Ser2P ChIP‐seq in CS‐B and HA CSB_WT_ cells. The sets generated contained 12,083 (H3K27me3), 33,390 (H3K27ac), and 63,687 (RNAPII Ser2P) unique peaks, respectively. For RNAPII Ser2P ChIP‐seq in HA CS‐B cells peak calling resulted in 17,941 UV and 19,567 + UV peaks. For ATAC‐seq data (Buenrostro et al., 2013), fragment size distribution plots were initially generated to ensure the quality of the data. Precomputed effective genome sizes (defined as the length of the “mappable” genome) were set accordingly, based on the examined organism and the respective genome build. Peak calling was utilised by both Genrich (v0.6.1, available at https://github.com/jsh58/Genrich) and MACS2. Genrich was used with the parameters j r e chrM v l 100 *p* 0.05 and MACS2 with the parameters‐f BAMPE‐fmin‐length 100. The results were merged to form the final peak sets, which were respectively 174,098 peaks for the UV condition, 176,755 peaks for the 30 min + UV, and 187,947 peaks for the 2 h + UV in CS‐B cells.

### 2.13 Annotation of regions/genes of interest

Regional distribution annotation of peaks was performed through Chipseeker (v1.22.1, (Yu et al., 2015)). The annotation includes the following regions, ordered in accordance with their priority for possible annotation overlaps: Promoter, 5’ UTR, 3’ UTR, Exon, Intron, Downstream (defined as downstream of gene end), Intergenic.

### 2.14 Promoter EI calculations

EI was calculated by taking the average coverage in rpm in the gene body (read density in the gene body (Db) from 100 bp to 2 kb downstream of TSS for genes larger than 2 kb) divided by the average coverage on the promoter‐proximal region (Dp, from −250 bp to +100 bp around TSSs). EI changes (ΔEI) between conditions were calculated by dividing various +UV conditions with the steady state condition (NO UV). The proportion of genes with increased EI after treatment are shown in percentages, and calculations took place with *coverageBed —counts* on the regions of interest, after transforming bams to beds with *bed2bam*.

### 2.15 GRO‐seq analysis

For GRO‐seq, reads were quality trimmed, and Illumina adaptors were removed with cutadapt, keeping resulting reads of at least 20 bp (cutadapt‐q and‐m parameters). To remove rRNA reads, QC‐filtered reads were first aligned to the human ribosomal DNA complete repeating unit (NCBI id: U13369.1) allowing up to 1 mismatch using Bowtie8 (version 1.1.0) (bowtie‐n‐k 1‐m 1 and—un parameters), and the unmapped reads were aligned uniquely to the GRCh37/hg19 reference genome allowing up to 2 mismatches (bowtie n 2k 1 and m 1 parameters).

Two gene length thresholds (100 kb and 250 kb) were used depending on the time point and condition. To generate elongation wave progression estimates on all time points (10 min, 20 min, 60 min, 120 min) of the +UV condition, as also for NO UV 10 min and 15 min, the first 100 kb of genes larger than 100 kb binned into 100 bp bins were used. For 60 min NO UV time point, the first 250 kb of genes larger than 250 kb were binned into 100 bp regions. Counts on bins were extracted using the intersect command of pybedtools and the corresponding samples for each annotation.

Elongation wave progression estimation was then performed as described in (Fanourgakis et al., 2023). Gene regions used in average profile and heatmap visualizations were cut to a fixed length and split into equally sized genomic bins using custom scripts. Counts on bins were calculated with pybedtools (Dale et al., 2011; Quinlan & Hall, 2010) intersect and were normalized for region size and library depth using the Reads Per Kilobase of transcript, per Million mapped reads (RPKM) formula using custom Python scripts and pandas package (McKinney, 2010). Average profiles were produced using custom Python scripts using Matplotlib's pyplot, and single‐sample heatmaps were produced by SeqMiner (Zhan & Liu, 2015). Boxplot plots were produced with seaborn package (Waskom, 2021).

### 2.16 Retention analysis using Pulse Chase data

Exonic and gene counts for all genes of the *TxDb.Hsapiens.UCSC.hg19.knownGene* annotation object were measured using the R package *GenomicFeatures* (v1.21.30, (Lawrence et al., 2013)), for all conditions of the Pulse Chase datasets in both CS‐B and VH10 cell lines (‐UV, +UV 1.5 h, 6.5 h, 8 h), and‐UV condition of Nascent data. Intronic counts were defined by subtracting the exonic from the gene measurements. Since the TxDb annotation holds all genes, and to create an active Pulse Chase list dependent only on itself, only genes with counts more than the median values of each condition were kept for further analysis, excluding inactive, suppressed, and/or noisy genes from subsequent calculations. After isolating the genes passing all filters and being present in all conditions of both cell lines and in both experimental setups (Pulse Chase, Nascent), we were left with 10,205 genes in number, which had a ~ 90% overlap with the CS‐B and VH10 active lists defined earlier. With this cohort, retainment measurements took place as follows: retention of a gene = Pulse Chase intronic counts at t1 / Nascent gene counts at t0. Library size normalization took place before calculations. The significance of differences among cell lines for each time point was measured by taking the 1st to 3rd quartile scores and calculating a 1000‐fold permutation‐based t‐test with sampling (100 instances).

### 2.17 Differential accessibility/enrichment analysis

Differential Accessibility analysis (DAR) for ATAC‐seq and Differential Enrichment Analyses for ChIP‐seq data was performed by utilizing *diffbind* (v2.14.0, https://www.bioconductor.org/packages//2.10/bioc/html/DiffBind.html) in *DESEQ2* mode (v1.26.0, (Love et al., 2014)). Differential H3K27ac ChIP‐seq in CS‐B cells: p‐value threshold = 0.05, Fold Change = 1. Differential ATAC‐seq analysis in CS‐B cells: p‐value threshold = 0.001, Fold change = 1.

### 2.18 Nucleosome positioning analysis

Nucleosome positioning analysis was performed with NucleoATAC (v0.3.4, (Schep et al., 2015)) for all ATAC‐seq conditions (NOUV, 10 min, 30 min), for CS‐B and VH10 cells, using the default parameters. All regions from the active TSSs to +250 bp downstream were analysed, targeting the first downstream nucleosome of genes, and then all regions from active TSSs to +1 Kb, to target both the +1 and + 2 dyads, if present. NucleoATAC‐R (v1.1) was used to create V‐plots, visualising the average + 1 dyad occupancy for each time point in both cell lines. The subtraction of the VH10 Vplot's matrix numbers (i.e. signal) from the CS‐B Vplot matrix for each shared time point, gives the true difference of signal density around the +1 dyad structures. All negative regions emerging from the subtraction were assigned with zeros. Statistical significance of the differences was measured through paired t‐test calculations on the vectors created from the flattening of the Vplot matrices involved (colSums). Fuzziness of the +1 nucleosome was also calculated with NucleoATAC (results file.*nucpos*.*bed*.*gz*). Fuzziness of the top high or low escaping genes (1000 highest and 1000 lowest) was examined after sorting the ΔEI of the genes in ascending order (30 min 20 J/m^2^ EI/ NO UV EI). *bedtools closest k 2* was used, to call the 2 closest (+1 and + 2 distance) neighboring dyad centers to each active TSS. Distances larger than 1 Kb were excluded, and of the remaining +1 and + 2 called neighbors, the most common distance (the density point with most occurrences—max) was taken as the expected for each cell line.

## 3 RESULTS

### 3.1 Release of RNAPII from PPP sites in CS‐B fibroblasts as an early event post‐UV treatment

**FIGURE 1:**
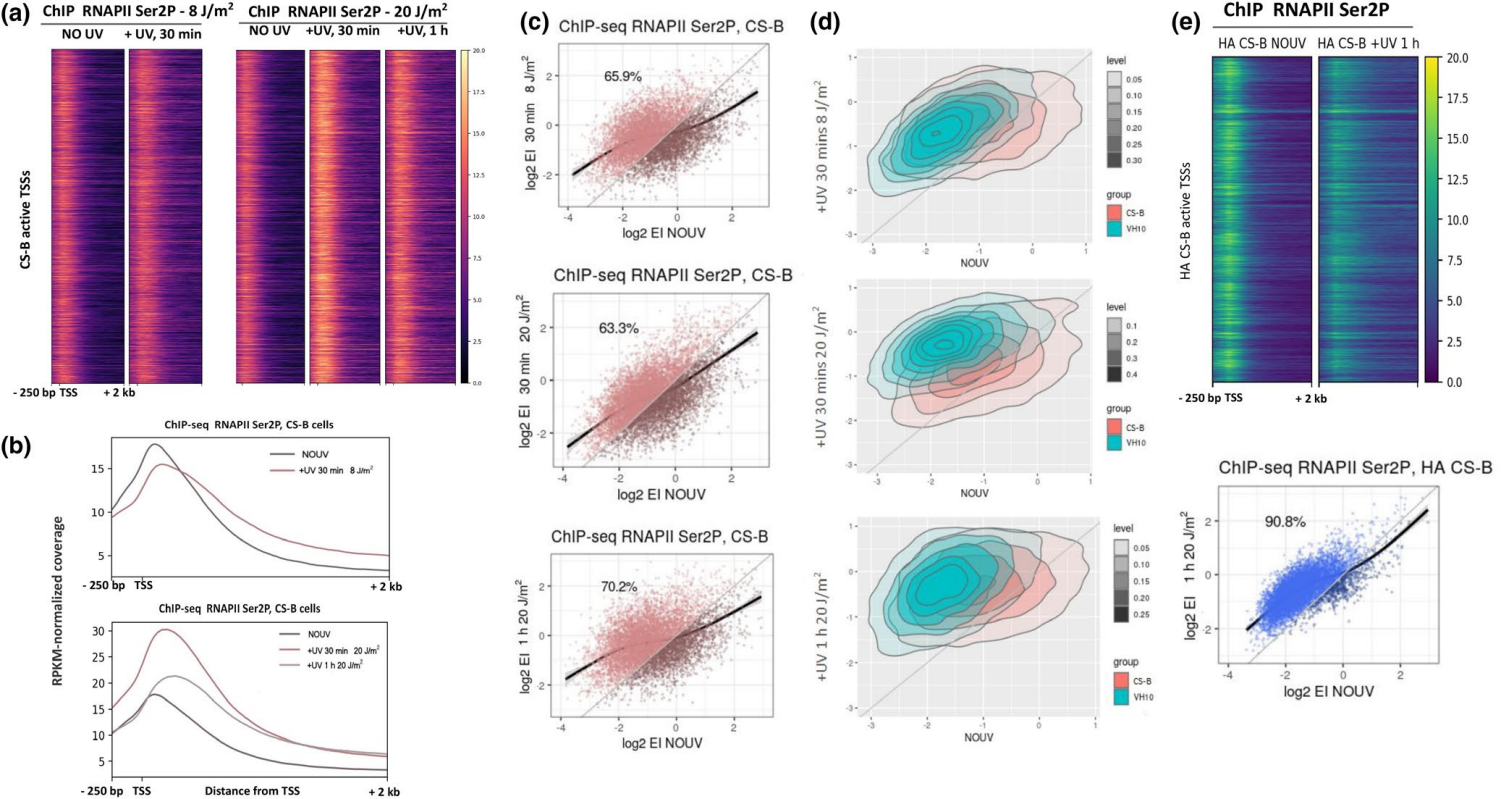
Release of RNAPII from PPP sites in CS‐B fibroblasts early after UV treatment. (a) Heatmap illustrating RNAPII‐ser2 ChIP‐seq signal on genomic regions −250 bp to +2 kb around active TSSs of non‐irradiated (NO UV) and irradiated CS‐B fibroblasts. (Upper) Irradiated cells were treated with 8 J/m^2^ UV dose and left to recover for 30 mins after exposure. (Lower) Irradiated cells were treated with 20 J/m^2^ UV dose and left to recover for 30 mins and 1 h after exposure, respectively. (b) Average profiles of RNAPII‐ser2 ChIP‐seq signal for the respective conditions of panel A. (c) Escape index (EI) analyses for RNAPII‐ser2 ChIP‐seq comparing control (NO UV) and irradiated ((Upper) UV 8 J/m^2^ and 30 mins recovery, (Middle) UV 20 J/m^2^ and 30 mins recovery, (Lower) UV 20 J/m^2^ and 1 h recovery) CS‐B fibroblasts. Percentages of loci with increased EI in each conditions are depicted and shown in lighter color. (d) Density plots showing the escape index (EI) of normal (VH10 htert) and CS‐B fibroblasts under the same conditions and timepoints, on a common active gene set (6498 genes). (e) (Upper) Heatmap illustrating RNAPII‐ser2 ChIP‐seq signal on genomic regions −250 bp to +2 kb around active TSSs of non‐irradiated (NO UV) and irradiated (+UV, 1 h, 20 J/m^2^) HA CS‐B fibroblasts. (Lower)) Escape Index (EI) analysis for RNAPII‐Ser2 ChIP‐seq comparing control (NO UV) and irradiated (+UV, 1 h, 20 J/m^2^) HA‐CSB fibroblasts.

As mentioned, exposure to UV triggers a multi‐layered transcriptional response, including the activation of P‐TEFb (Bugai et al., 2019; Chen et al., 2008) and the fast release of PPP RNAPII molecules in the gene bodies of actively transcribed genes (Borisova et al., 2018; Lavigne et al., 2017; Liakos et al., 2020). This response results in accelerated repair in NER‐proficient cells and shapes the mutational landscape of genotoxic exposed cancer tissues (Lavigne et al., 2017; Liakos et al., 2023). As RNAPII PPP release occurs early after damage induction and throughout the transcribed genome in TC‐NER proficient cells (Liakos et al., 2020), we questioned whether a similar response takes place in TC‐NER deficient CS‐B cells following exposure to UV damage. We performed ChIP‐seq experiments against the elongating RNAPII (hyperphosphorylated RNAPII at Ser2 (Ser2P), see Figure S1a for Pearson Correlation coefficients) and calculated EI ratios (Figure S1b, ChIP‐seq reads at gene body/ChIP‐seq reads at the promoter, see also Methods for details) for irradiated and non‐irradiated control cells. Interestingly, we observed a shift of RNAPII Ser2P molecules in regions downstream of TSS after exposure to different doses of UV irradiation (Figure 1a,b), leading to an increase in EI (Figure 1c). Specifically, 65.9% of genes showed an increase of EI 30 min after exposure to 8 J/m^2^ UV irradiation (Figure 1c, upper), while 63.3% and 70.2% of genes followed the same trend when examined at 30 min and 1 h after exposure to 20 J/m^2^, respectively (Figure 1c, middle and lower). The shift of RNAPII in regions downstream of PPP sites was also confirmed through ChIP‐qPCR on various DNA loci and upon different doses of UV (8 J/m^2^‐20 J/m^2^) (Figure S1c,d). These findings indicate that similar to their normal counterparts, UV exposure results in the prompt release of paused RNAPII in the gene bodies of CS‐B fibroblasts, in most active genes. However, we noticed that the fraction of genes exhibiting increased EI in CS‐B fibroblasts was significantly smaller compared to that of normal fibroblasts (89%–91%) (Figure S2a, data obtained from Lavigne et al., 2017). Notably, density plots of the EI for a cohort of commonly active genes between the normal and CS‐B hTert cell lines (6.498 genes) demonstrated a more pronounced post‐UV increase of EI for normal fibroblasts compared to CS‐B counterparts (Figure 1d). To validate the globality of RNAPII PPP release in TC‐NER proficient cells in comparison to the post‐UV EI of RNAPII that we observed in CS‐B hTert cells, we performed RNAPII Ser2P ChIP‐seq in the same parental CS‐B deficient cell line (CS1AN‐SV40 immortalised) that express HA‐tagged wild‐type CSB protein (HA CSB_wt_) (Van Gool et al., 1997) (Figure 1e (Upper), Figure S2b). Similar to VH10 cells, the vast majority of active genes (90.8%) in HA CSB_wt_ cells showed enhanced release of RNAPII into elongation after exposure to UV (Figure 1e (Lower)). We concluded that similar to normal cells, UV‐exposed CS‐B fibroblasts release elongating RNAPII in the bodies of active genes during the first hours after damage induction, demonstrating that this response is independent of the ability of the cells to perform TC‐NER. However, the globality and the magnitude of this UV transcriptional response are weakened in CS‐B fibroblasts.

### 3.2 Comparison of transcription elongation speed in normal and CS‐B fibroblasts in steady state and upon UV

**FIGURE 2:**
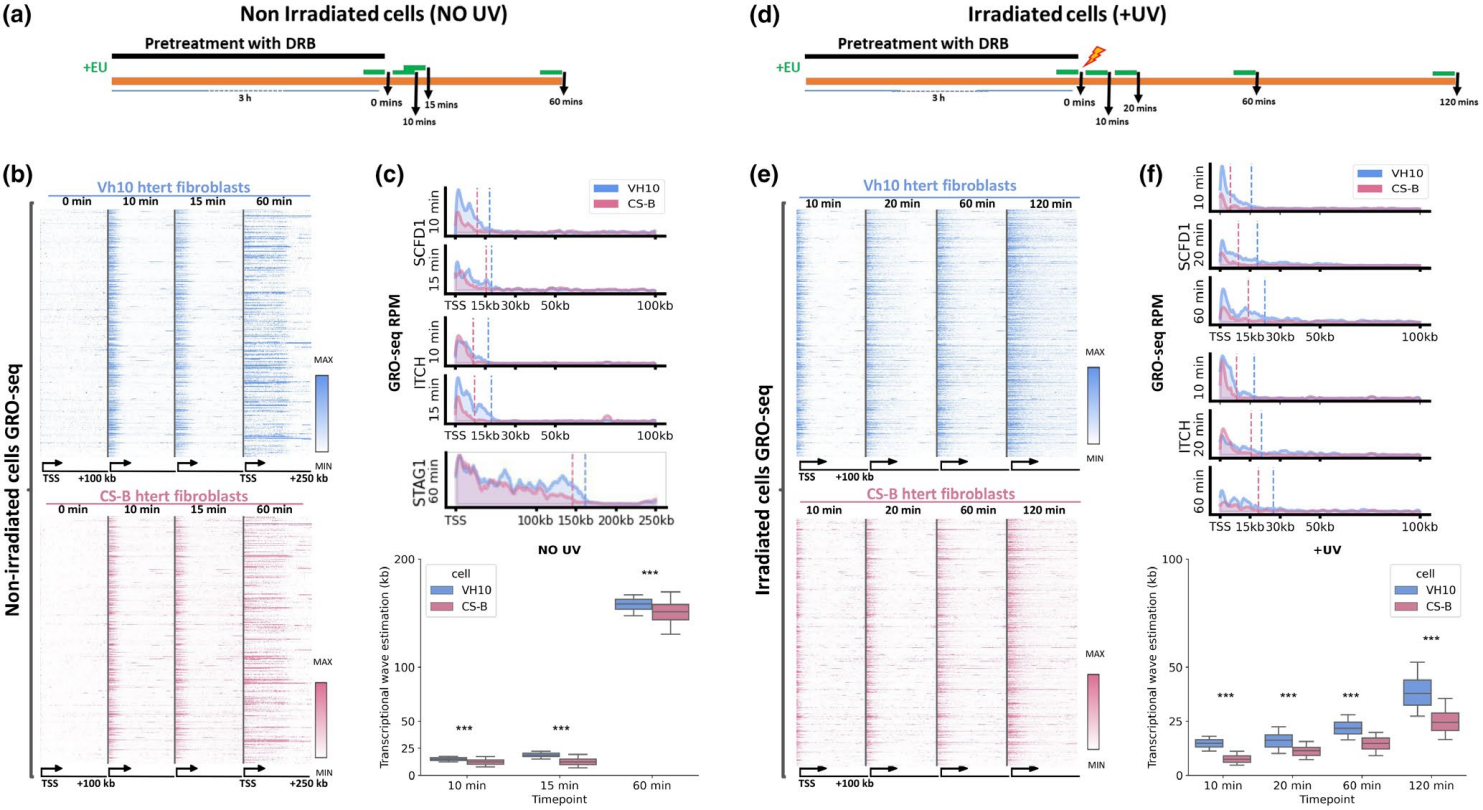
Comparison of transcription elongation speed in normal and CS‐B fibroblasts in steady state and upon UV. (a) Experimental timeline depicting DRB/GRO‐seq experimental setup in non‐irradiated normal (VH10 htert) and CS‐B htert skin fibroblasts. EU labeling periods are indicated in green. (b) Heatmaps depicting GRO‐seq signal and transcription wave progression at the indicated time points for non‐irradiated Vh10 htert (upper) and CS‐B htert (lower) skin fibroblasts. Depicted genomic regions are indicated. (c) (Upper) Density plot of GRO‐seq signal at *SCFD1*, *ITCH* and *STAG1* genes, respectively, at the indicated time points in non‐irradiated VH10 htert (blue) and CS‐B htert (pink) skin fibroblasts, dashed lines indicate the estimated length of the transcriptional wave. (Lower) Progression of transcriptional waves based on Hidden Markov Model (HMM) for the two cell lines, in the indicated time points for non‐irradiated Vh10 htert and CS‐B htert skin fibroblasts. Asterisks (*) show the paired t‐test significance of the difference between the two cell lines for the time points indicated. (d) Experimental timeline depicting DRB/GRO‐seq experimental setup in irradiated (15 J/m^2^) normal (VH10 htert) and CS‐B htert skin fibroblasts. EU labeling periods are indicated in green. (e) Same as in b but for irradiated VH10 htert and CS‐B htert skin fibroblasts. Depicted genomic regions are indicated. (f) Same as in c but for irradiated VH10 htert and CS‐B htert skin fibroblasts.

The above findings suggested that similar to wild‐type cells (Lavigne et al., 2017; Liakos et al., 2020; van den Heuvel et al., 2021), UV exposure triggers a wave of nascent transcription in CS‐B fibroblasts. To examine the kinetics of this phenomenon in a more resolutive fashion, we performed GRO‐seq experiments in non‐irradiated and irradiated CS‐B fibroblasts and compared them with normal counterparts (Figure 2a). Specifically, before UV treatment cells were treated with DRB, a widely used transcription inhibitor that prevents the release of elongating RNAPII from PPP sites. This treatment resulted in the clearance of gene bodies from actively elongating RNAPII molecules and nascent transcripts before the exposure of cells to UV. After UV treatment, cells were recovered in their normal growing medium (without DRB) and the progression of the nascent transcription wave that was generated was defined in each cell line (VH10 vs. CS‐B) and condition (NO UV vs. UV), respectively (see Methods for details). As expected for non‐irradiated cells of both cell lines tested transcription progressed faster, reaching distal regions of long gene bodies within 1 h after release from DRB (Figure 2b,c). Interestingly, the transcription wave was found to progress faster in normal compared to CS‐B fibroblasts (Figure 2c, pairwise‐Pearson *p*‐value <0.001 (***) for 10′ and 15′ and 60′ time points), suggesting a possible general role for CSB protein in facilitating transcriptional elongation. The speed of transcription elongation was reduced upon UV exposure for normal cells, but comparably, this delay was amplified in CS‐B fibroblasts (Figure 2d–f, pairwise‐Pearson p‐value <0.001 (***) for all time points tested). In addition, a similar delay in transcription progression was noticed in irradiated CS‐B SV40 fibroblasts compared to irradiated HA CSB_wt_ (Figure S3a–c). Given the role of CSB protein in TC‐NER initiation, we believe that, in addition to the potential role of CSB in elongation, this significant delay of the transcription wave progression derives from the prolonged stalling of RNAPII at damaged sites due to the inability of CS‐B cells to resolve the UV‐induced lesions.

### 3.3 Retainment of incomplete nascent transcripts in CS‐B cells after UV irradiation

**FIGURE 3:**
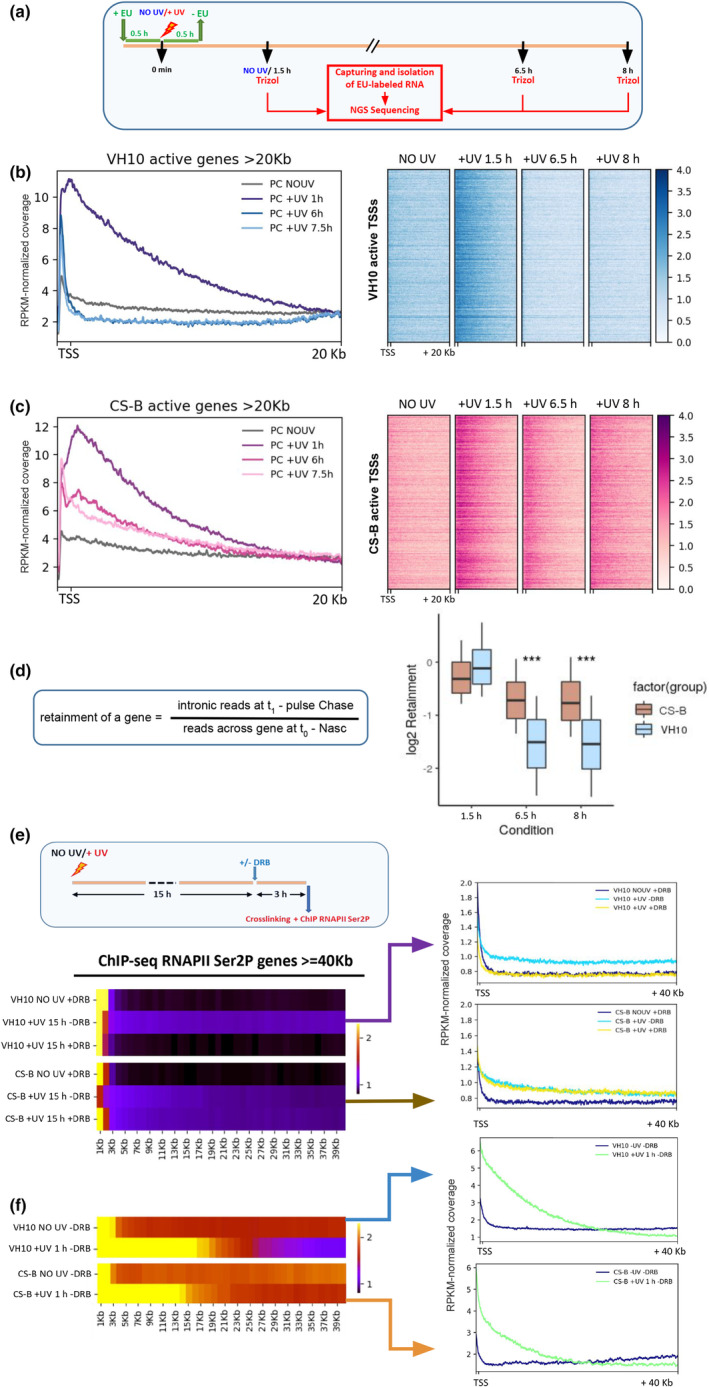
Retainment of unprocessed nascent transcripts in CS‐B cells after UV irradiation. (a) Experimental timeline of Pulse/Chase experiment performed in VH10‐htert and CS‐B htert skin fibroblasts. (b) Average profile (left) and heatmap (right) of the Pulse‐Chase‐seq experiments in the Vh10‐htert fibroblasts, in genomic regions −250 to +20 kb around TSSs of active Vh10‐htert genes. (c) Same as in b, but for CS‐B skin fibroblasts in genomic regions −250 to +20 kb around TSSs of active CS‐B htert genes. (d) Retainment ratios of commonly active genes for the CS‐B and VH10 htert fibroblasts. Extreme values were excluded (1st–3rd quantile values used). Asterisks (*) indicate a p‐value significance of a permutation based t‐test among cell lines. (e) Experimental timeline. (Upper) Heatmap of RNAPII Ser2P ChIP‐seq density in genomic bins of 1 kb for VH10 and CS‐B fibroblasts treated as depicted in the experimental timeline (Upper panel). Regions from TSS to +40 kb are depicted in active genes larger than 40 kb. (f) Heatmap of RNAPII Ser2P ChIP‐seq in genomic bins of 1 kb for non‐irradiated (NO UV DRB) and irradiated (+UV, 1 h, DRB) VH10 and CS‐B fibroblasts. Data for Vh10 fibroblasts were obtained from Lavigne et al., 2017.

Our results so far indicated that, although slower in progression compared to normal cells, de novo transcriptional waves are detectable in CS‐B fibroblasts after UV irradiation. However, the fate of the concomitant, newly synthesized UV‐triggered transcripts and whether they are handled differently in normal and CS‐B fibroblasts has not been examined thus far. To address this, we conducted sets of Pulse‐Chase experiments in normal and CS‐B fibroblasts (Figure 3a; Figure S4a,b, see Methods for details**)**. Briefly, cells were labelled with EU for 1 h, half an hour prior and half an hour post UV irradiation, before EU was removed and cells were left to recover for the indicated time, as depicted in Figure 3a. Total RNA was collected and the EU‐labelled nRNA was captured, isolated and analysed by NGS. Genes with extremely low Pulse Chase signal were excluded from further analysis in each cell line (Figure S4c, see also Methods). Our results showed that in normal cells the initially labelled nRNA (nRNA that was being transcribed normally the last 30 min before and 30 min after exposure of the cells to UV) was detected throughout the first 20 kb of gene bodies at 1.5 h after UV. In contrast, at 6.5 h and 8 h post UV irradiation, its signal was significantly reduced (Figure 3b). This was not the case for the irradiated CS‐B fibroblasts where the initially labelled RNA could still be detected even 8 h after UV (7.5 h after removal of EU, Figure 3c). To acquire a quantitative view of this phenomenon, we calculated a retention metric for each transcript. This was calculated as the Pulse‐Chase intronic reads at the time point of interest (1 h, 6.5 h, 8 h) divided by the nRNA‐seq signal for this transcript, in non‐irradiated (control) conditions (Figure 3d, See also Methods). Indeed, irradiated CS‐B fibroblasts showed increased retention of nascent transcripts at 6.5 h and 8 h after UV treatment when compared to TC‐NER proficient cells (Figure 3d). These findings suggest that in UV‐exposed CS‐B fibroblasts either nRNAs remain bound to damage‐arrested RNAPII molecules for many hours or their increased detection consists of a reflection of a general defect for RNA degradation machinery in UV‐treated CS‐B fibroblasts.

Subsequently, we aimed to investigate whether elongating (RNAPII Ser2P) molecules exhibit a corresponding retention pattern in irradiated CS‐B fibroblasts, compared to wild‐type equivalents (VH10 htert cells). To this end, we irradiated CS‐B cells with UV and applied DRB at 2 h after damage exposure, for 2 h (Figure S5a). We noted an augmented retention of RNAPII Ser2P in chromatin of respective extracts in irradiated CS‐B fibroblasts, in earlier time points after damage induction (up to 4 h after UV exposure), compared to normal cells (Figures S5b–d, compare levels of RNAPII Ser2P before (+UV, DRB (2 h)) and 2 h after (+UV, +DRB (4 h)) the addition of DRB).

To examine this further at later time points after exposure to UV damage, we conducted the experimental timeline depicted in Figure 3e. Briefly, normal and CS‐B fibroblasts were subjected (or not) to UV treatment, and after 15 h DRB was applied (or not) for 3 h. Cells in each experimental condition were then crosslinked (18 h post‐UV) and ChIP‐seq against RNAPII Ser2P was performed. Similar to their non‐irradiated normal counterparts, the inhibition of pause‐release through DRB led to a depletion of RNAPII Ser2P from gene bodies in non‐treated CS‐B cells (Figure 3e, see heatmap and average profiles) (NO UV—DRB vs. NO UV + DRB), for VH10 and CS‐B cells. The same outcome was observed when DRB was applied to irradiated normal cells (Figure 3e, see +UV 15 h + DRB for VH10 cells (heatmap and Upper Average profile)) whereas a small number of transcribing RNAPII molecules was detected in the absence of DRB (Figure 3e, see +UV 15 h—DRB for VH10 cells (heatmap and Upper Average Profile)). However, in UV‐treated CS‐B fibroblasts, the levels of chromatin‐bound RNAPII Ser2P remained essentially unchanged before and after DRB treatment (Figure 3e, see +UV 15 h −/+ DRB in CS‐B cells (heatmap and Lower Average profile)). A comparison of elongating RNAPII signal between Vh10 and CS‐B cells of NO UV and + UV 1 h time points in the absence of DRB (Figure 3f) shows the increased retainment of prior‐to‐UV elongating RNAPII in actively transcribed genes in CS‐B cells. Collectively, our results show that apart from the elevated presence of nascent transcripts, irradiated CS‐B fibroblasts were also characterized by increased retainment of RNAPII Ser2P in gene bodies in both the early and late phase of UV recovery, compared to UV‐treated normal fibroblasts.

### 3.4 Continuous de novo recruitment of initiating RNAPII (RNAPII‐hypo) takes place during the early stages of recovery from UV exposure in CS‐B fibroblasts

**FIGURE 4:**
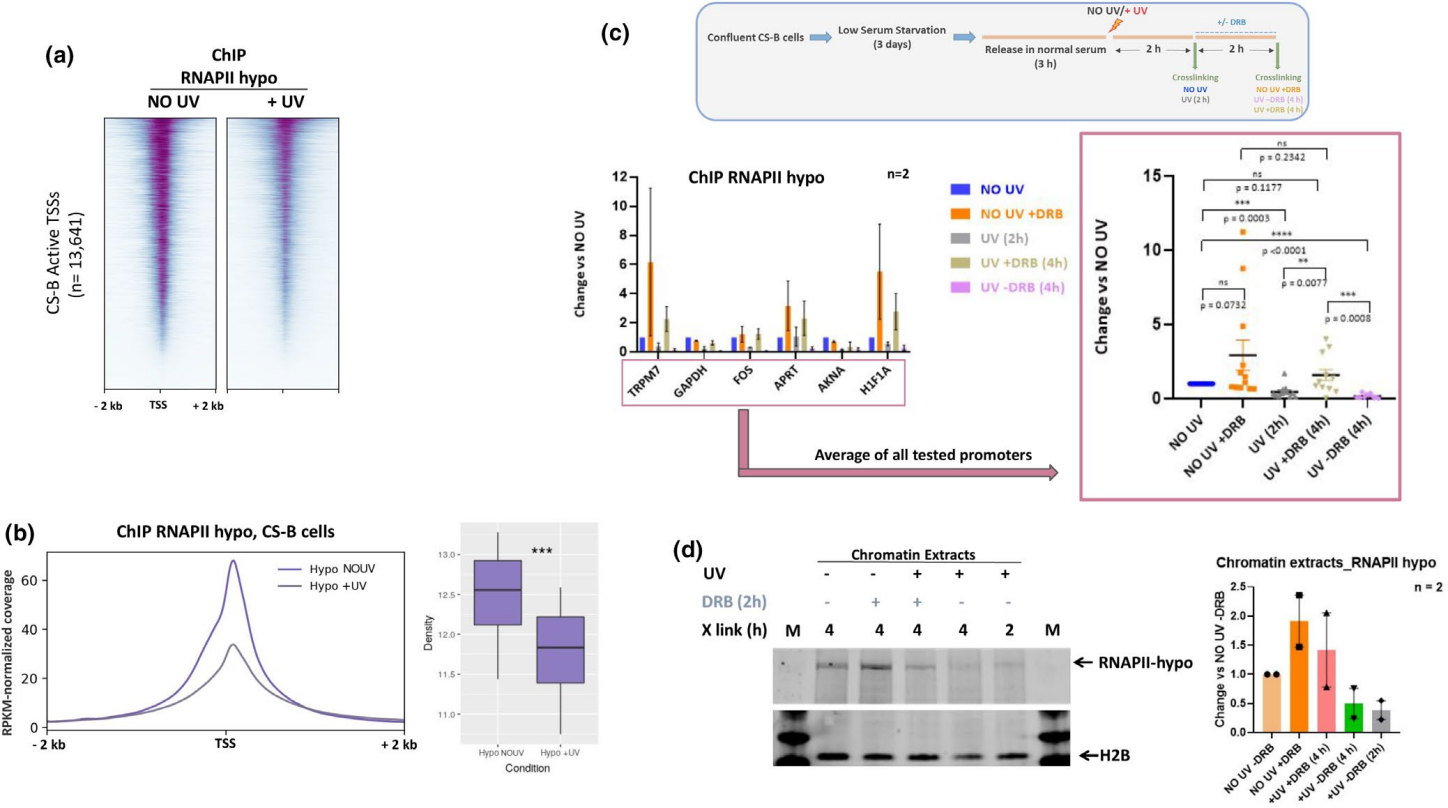
De novo recruitment of initiating RNAPII during the early stages of UV‐recovery in CS‐B fibroblasts. (a) Heatmap depicting RNAPII‐hypo ChIP‐seq signal 2 kb around of active TSSs of non‐irradiated (NO UV) and irradiated (+UV (15 J/m^2^), 2 h) CS‐B htert fibroblasts. (b) (Left) Average profiles for RNAPII‐hypo ChIP‐seq signal at active TSSs of non‐irradiated (NO UV) and irradiated (+UV (15 J/m^2^), 2 h) CS‐B htert fibroblasts. (Right) Boxplots of the 1st to 3rd quantile values from the RNAPII‐hypo ChIP‐seq signal 2Kb around active TSSs and a permutation based t‐test (*1000, pearson) on the significance of the drop after irradiation. (c) (Upper) Experimental timeline. Irradiated cells were treated with 15 J/m^2^. (Lower, Left) ChIP qPCR results depicting RNAPII‐hypo enrichment in selected promoter regions of active genes. Fold changes were normalized to a negative primer (ChIA neg) and then expressed as compared to control cells (NO UV). Error bars represent standard error of the mean (S.E.M) (Lower, Right) Average ChIP qPCR enrichment for all tested promoters. For each condition individual experiments are depicted as dots with respective color and symbol (Error bars represent S.E.M.). P values of unpaired *t*‐tests are indicated. (d) (Left) Western Blot of crosslinked chromatin extracts in CS‐B htert fibroblasts, treated as explained in C (Upper). H2B was used as loading control. (Right) Quantification of RNAPII‐hypo levels as compared to control condition (NO UV, − DRB). Results were obtained from two independent biological replicates (*n* = 2). Error bars represent standard error of the mean (S.E.M.).

Depletion of chromatin‐bound pre‐initiating RNAPII (hypo‐phosphorylated) following UV exposure (Heine et al., 2008; Rockx et al., 2000; Tufegdžić Vidaković et al., 2020) was historically considered an early feature of transcriptional repression for new initiation events, in both repair proficient and deficient fibroblasts. Nevertheless, recent studies in normal fibroblasts (Bay et al., 2022; Liakos et al., 2020, 2023) provided new evidence suggesting that de novo transcription initiation and the transition to elongation are enhanced during the early recovery period from UV stress. In the present study, the aforementioned findings show that there is a UV‐triggered release of RNAPII molecules from PPP sites in CS‐B cells. However, it remains unclear whether this phenomenon is sustained through the continuous recruitment of pre‐initiating RNAPII at promoters and transcription initiation. In our hands, ChIP‐seq experiments against the RNAPII‐hypo isoform (hypo‐phosphorylated RNAPII) in CS‐B fibroblasts (Figure S6a) showed decrease of the signal at regions close to TSS 2 h after UV irradiation (15 J/m^2^) (Figure 4a,b), confirming previous observations in CS cells (Kristensen et al., 2013; Proietti‐De‐Santis et al., 2006). To examine whether this drop of RNAPII‐hypo following exposure to UV was due to the gradual elimination of RNAPII‐hypo assembly at active promoters of CS‐B cells or was caused by increased promoter escape and transition to elongation similar to wild‐type cells (Liakos et al., 2020), we performed a set of time‐ and transcription inhibitor‐resolved experiments. Briefly, irradiated CS‐B cells were left to recover for 2 h after UV and then DRB, an inhibitor of RNAPII release from PPP sites, was applied, or not. Subsequently, cells were incubated for another 2 h, before crosslinking. Non‐irradiated CS‐B cells were also used as controls and were crosslinked at the same time points according to the experimental timeline (Figure 4c). Crosslinked cells of each experimental condition were subjected to ChIP against RNAPII‐hypo (see also Methods) and then the enrichment of RNAPII‐hypo binding for various promoters was assessed through qPCR. Strikingly, we observed that inhibition of pause‐release resulted in “rescue” of RNAPII‐hypo levels, in all promoters tested (Figure 4c). This trend of “rescue” was also evident in respective total chromatin extracts (Figure 4d). Together, these results demonstrate that (i) the drop of RNAPII‐hypo in active promoters of CS‐B fibroblasts after UV exposure is triggered by an enhanced, compared to steady‐state, PPP‐release and (ii) de novo recruitment of RNAPII‐hypo takes place throughout the early recovery period (up to 4 h) from UV‐induced genotoxic stress in CS‐B cells.

### 3.5 Assessment of chromatin alterations in UV‐treated CS‐B fibroblasts

**FIGURE 5:**
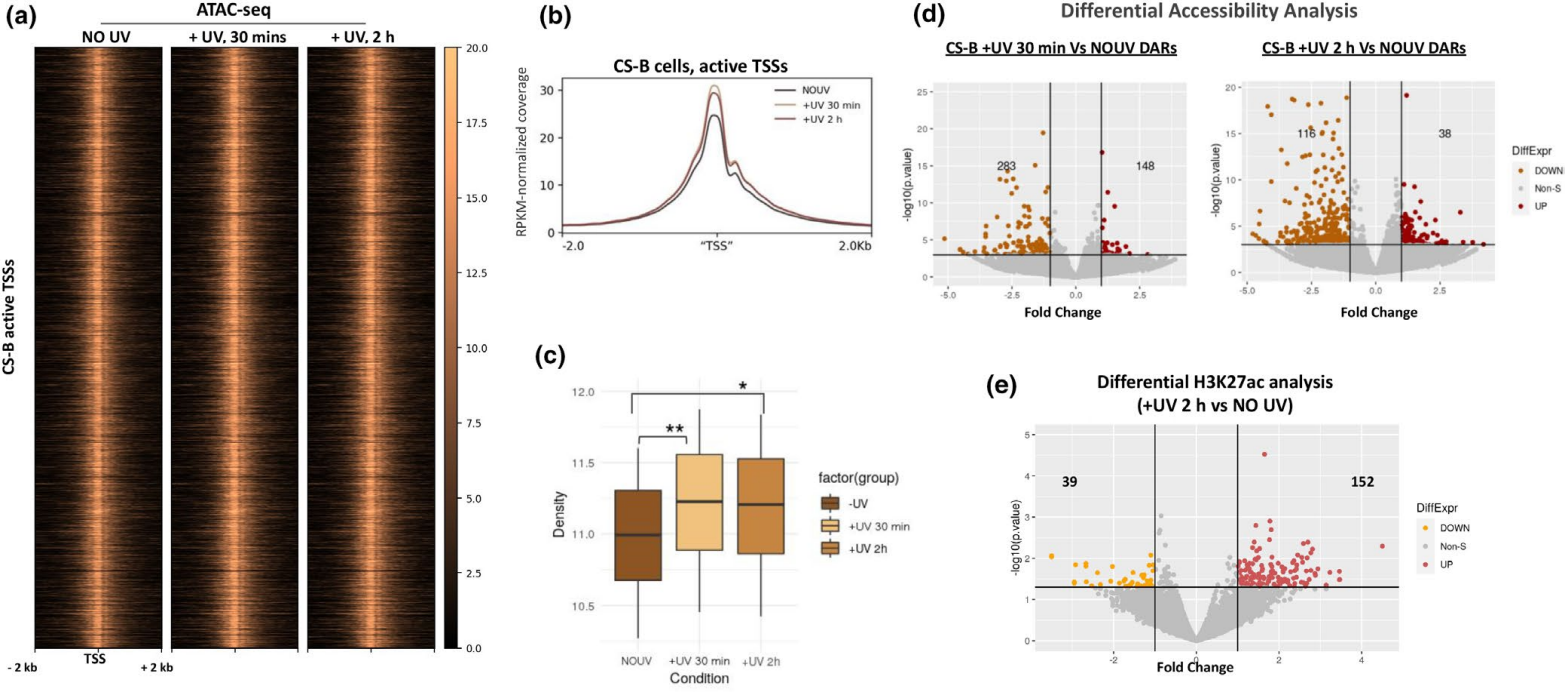
Limited changes in chromatin of UV‐treated CS‐B fibroblasts. (a) Heatmap showing ATAC‐seq signal at genomic regions 2 kb around of active TSSs of non‐irradiated (NO UV) and irradiated (+UV, 30 min, 2 h) CS‐B htert fibroblasts. (b) Average profiles depicting ATAC‐seq signal at genomic regions 2 kb around active TSSs of non‐irradiated (NO UV) and irradiated (+UV, 30 min, 2 h) CS‐B htert fibroblasts. (c) Boxplots of the 1st–3rd quantile values from the CS‐B ATAC‐seq signal 2Kb around active TSSs and a permutation based t‐test (*1000, Pearson correlation) to calculate the significance of the difference between non‐irradiated and irradiated conditions (NO UV Vs + UV 30 min *p*‐value = 0.009, NO UV Vs + UV 2 h p‐value = 0.04). (d) Differential accessibility analysis between non‐irradiated (NO UV, UV) and irradiated (+UV, 30 min (Left), +UV, 2 h (Right)) CS‐B cells. Genomic regions with significantly increased (dark red) or decreased (brown) accessibility are depicted, and their numbers for each comparison are indicated (p‐value threshold = 0.001, FC = 1). (e) Differential H3K27ac ChIP‐seq binding analysis between non‐irradiated and irradiated (+UV, 2 h, dose: 15 J / m^2^) CS‐B cells. Genomic regions showing increased (red) or decreased (yellow) targeting of H3K27ac after exposure to UV, are depicted (*p*‐value threshold = 0.05, FC = 1).

To investigate the impact of UV damage on the epigenome of CS‐B fibroblasts, we assessed chromatin accessibility through replicated ATAC‐seq experiments (Figure S7a) in non‐irradiated and irradiated cells, at 0.5 h and 2 h after exposure to UV. Fragment size distribution of our paired‐end sequenced libraries showed the expected periodical pattern ensuring the good quality of the experiments (Figure S7b). First, we examined chromatin accessibility at active promoters of CS‐B fibroblasts. We found that the signal increased in irradiated cells (in both 0.5 h and 2 h after UV), compared to non‐irradiated counterparts (Figure 5a–c). To identify significantly accessible regions in each experimental condition, we used MACS2 and Genrich peak‐calling algorithms and kept their union of peaks (See Methods). Using this approach we identified 174,098 peaks in non‐irradiated cells, while the number of detected regions increased to 176,755 and 187,947, for irradiated cells 0.5 h and 2 h, post‐UV, respectively (Table S5). Functional annotation of peaks showed that at steady state the majority of peaks were detected in regions close to promoters, introns, and distal intergenic loci (Figure S7c). No major differences were observed in the distribution of the detected ATAC‐seq peaks before and after exposure to UV (Figure S7c). An overall increase in the signal intensity of peaks was also evident, mostly at promoters (signal increase in 84.7% and 83.1% of peaks, at 0.5 h and 1 h, respectively) and intragenic regions (signal increase in 64% and 71.8% of peaks, at 0.5 h and 1 h, respectively) (Figure S8a–c). However, differential accessibility analysis revealed a limited number of genomic regions showing significantly increased or decreased signal (Figure 5d; Figure S8d), suggesting general stability for chromatin accessibility after UV exposure in CS‐B fibroblasts. This result was in contrast to findings showing a more robust opening of chromatin accessibility following UV exposure in normal fibroblasts (Liakos et al., 2020; Liu et al., 2021; Figure S9a). Next, we chose to examine the distribution of H3K27ac before and after UV exposure in CS‐B cells, a characteristic histone modification that demarcates active promoters and enhancers. For this reason, we performed H3K27ac ChIP‐seq experiments in non‐irradiated and irradiated (2 h after UV) CS‐B fibroblasts (Figure S9b) and performed peak calling to identify significant H3K27ac‐enriched loci (Table S6). Differential H3K27ac enrichment analysis between irradiated and control CS‐B cells revealed a small number of differentially bound loci (Figure 5e; Figure S9c). These observations were consistent with results from Western Blot analysis of bulk histone extracts in CS‐B fibroblasts, showing that total levels of H3K27ac remained essentially unchanged in the first 4 h after UV treatment (Figure S9d). Collectively, our data suggest that no major changes occurred in chromatin features correlated to active transcription in CS‐B cells, shortly after UV irradiation.

### 3.6 Nucleosome positioning analysis reveals a less dynamic and more compact chromatin structure in CS‐B fibroblasts

Transcription is a process occurring in the context of chromatin and as such depends on the positioning of nucleosomes and the degree of packaging of DNA around the histone octamer. Specifically, the positioning of the +1 nucleosome, the first nucleosome downstream of TSS, has been found to regulate the pausing of RNAPII at PPP sites (Jeronimo et al., 2021; Jimeno‐González et al., 2015). As shown above, exposure to UV triggers the de novo release of elongating RNAPII molecules from PPP sites in both CS‐B and HA CSB_wt_ fibroblasts in most active genes, a response previously seen also for UV‐exposed normal cells. However, the magnitude of this response and the fraction of genes responding were different between the two TC‐NER proficient and the CS‐B cells (see Figure 1; Figure S2a). We thus aimed to examine whether the positioning of the +1 nucleosome has a role in the aforementioned molecular mechanisms.

**FIGURE 6:**
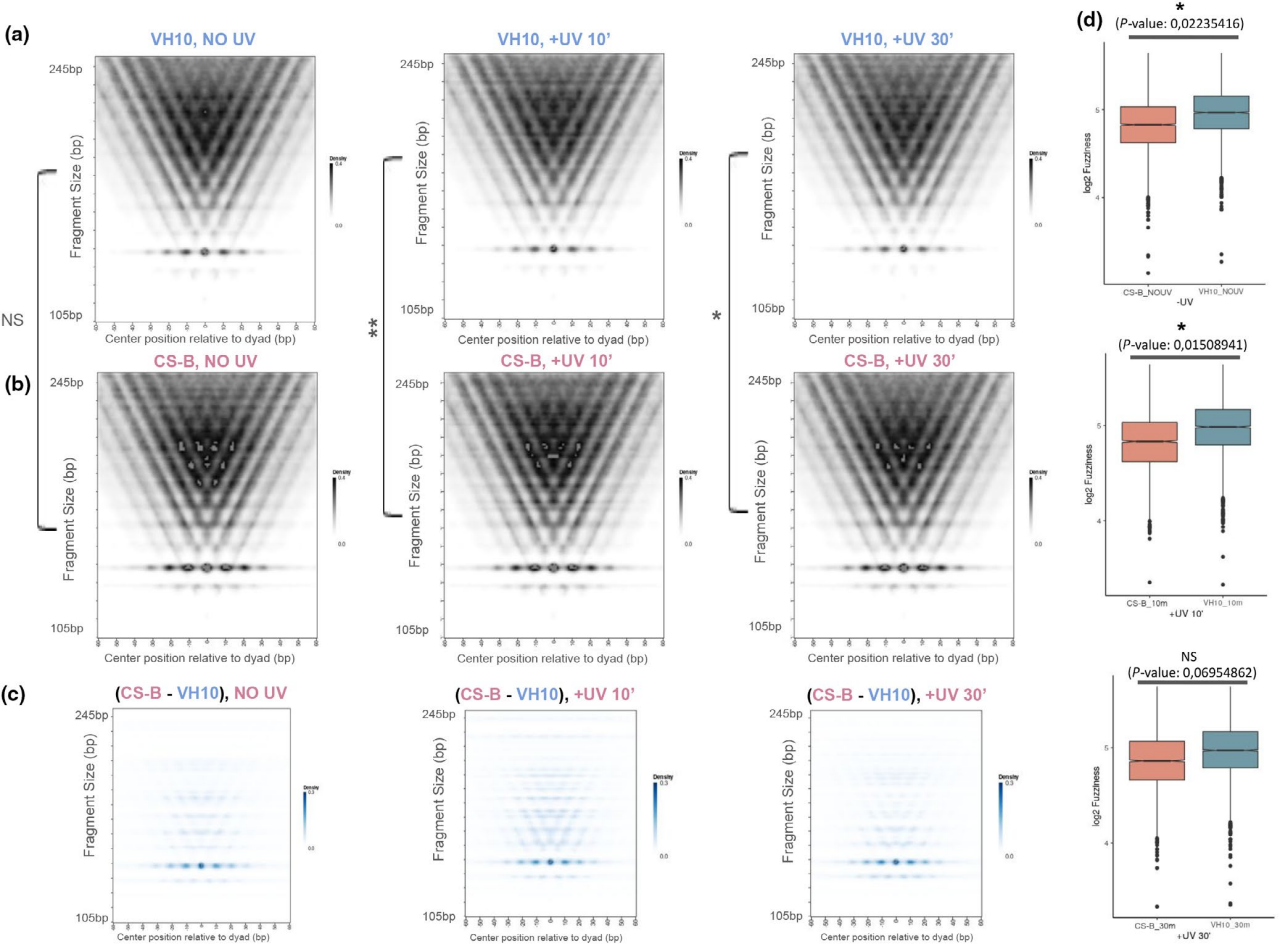
Comparison of nucleosome positioning in CS‐B and normal fibroblasts. (a) NucleoATAC Vplot results showing ATAC‐seq fragment signal, centered at +1 dyads in non‐irradiated (NO UV) and irradiated (+UV 10′, +UV 30′) VH10 htert cells. Vplots depict the density of specific fragment sizes in a position of interest (here, the +1 nucleosome as called by NucleoATAC). The nucleosomic dyad is centered on the X axis, and mononucleosome fragments can be detected ~147 bp vertically (dotted region). Larger ATAC‐seq fragments span mono‐, di‐ and poly‐nucleosomes. (b) Same as in a, but for CS‐B htert fibroblasts. Asterisks at the side of V plots indicate statistical significance (Wilcoxon *t*‐test) of the difference in mononucleosome abundance among cell lines. NS, non‐significant, *p*‐value <0.05 (*), *p*‐value <0.01(**). (c) Vplots showing signal after subtracting VH10 from CS‐B signal for each indicated timepoint. (d) Box plots depicting log2 fuzziness score difference of +1 dyads called by NucleoATAC for each cell line, in non‐irradiated (top, Wilcoxon's t‐test, *p*‐value: 0,02235416) and irradiated fibroblasts 10 min (middle, Wilcoxon's *t*‐test, *p‐*value: 0,01508941) and 30 min (bottom, Wilcoxon's *t*‐test, *p*‐value: 0,06954862) after exposure to UV.

To observe the immediate changes in nucleosome positioning after irradiation, we conducted additional sets of paired‐end ATAC‐seq experiments and examined genomic regions from the TSS of active genes to +250 bp, for mononucleosome dyads in non‐irradiated (NO UV) and irradiated (10 min and 30 min after damage induction) normal and CS‐B fibroblasts. (See Figure S10a for correlation plots). We used NucleoATAC, a well‐established Python package that estimates nucleosome dyad positions, and plotted the density of our ATAC‐seq fragments around +1 nucleosomes (Vplots, See also Methods). Our data showed a prevalence of mononucleosome‐spanning fragments for CS‐B fibroblasts, in all time points tested, when compared to normal counterparts (Figure 6a,b). At steady state, the difference between normal and CS‐B fibroblasts was very close to significance (Wilcoxon t‐test, p‐value: 0.05200806), while a more significant variation across cell lines was noticed for the +UV conditions at 10 min (Wilcoxon t‐test, *p*‐value: 0.001887166) and 30 min (Wilcoxon t‐test, p‐value: 0.01343623) after exposure to UV, respectively. This was also evident when subtracting the VH10 from the CS‐B mononucleosome signal for each experimental condition (Figure 6c). The respective fragment density plots corroborated these observations showing that the frequency of nucleosome‐spanning fragments is higher in CS‐B fibroblasts compared to normal counterparts for all experimental conditions (Figure S10b,c).

Next, we calculated the fuzziness score (inferred by NucleoATAC) for each sample in genomic regions ranging from active TSS to +250 bp, measuring the positioning uncertainty for +1 nucleosomes. Strikingly, the fuzziness score for VH10 fibroblasts was significantly higher compared to CS‐B in steady state conditions, as well as at 10 min after UV exposure (Figure 6d). Finally, we assessed the spacing between nucleosomes per cell line and experimental condition. Specifically, we focused on the distances of +1 and + 2 nucleosomes in control and irradiated normal and CS‐B fibroblasts, from their respective TSS. Notably, we observed that while inter‐dyad distance increases in normal fibroblasts after exposure to UV, this is hardly noticeable in CS‐B fibroblasts (Figure S10d). Collectively, our data suggest that in the absence of a functional CSB protein, +1 nucleosomes are positioned more firmly, their remodeling in response to DNA damage is delayed and the chromatin structure downstream of active TSSs is more compact compared to normal conditions.

### 3.7 Shared genomic occupancy for CSB and RNAPII in promoter‐proximal regions of active genes

**FIGURE 7:**
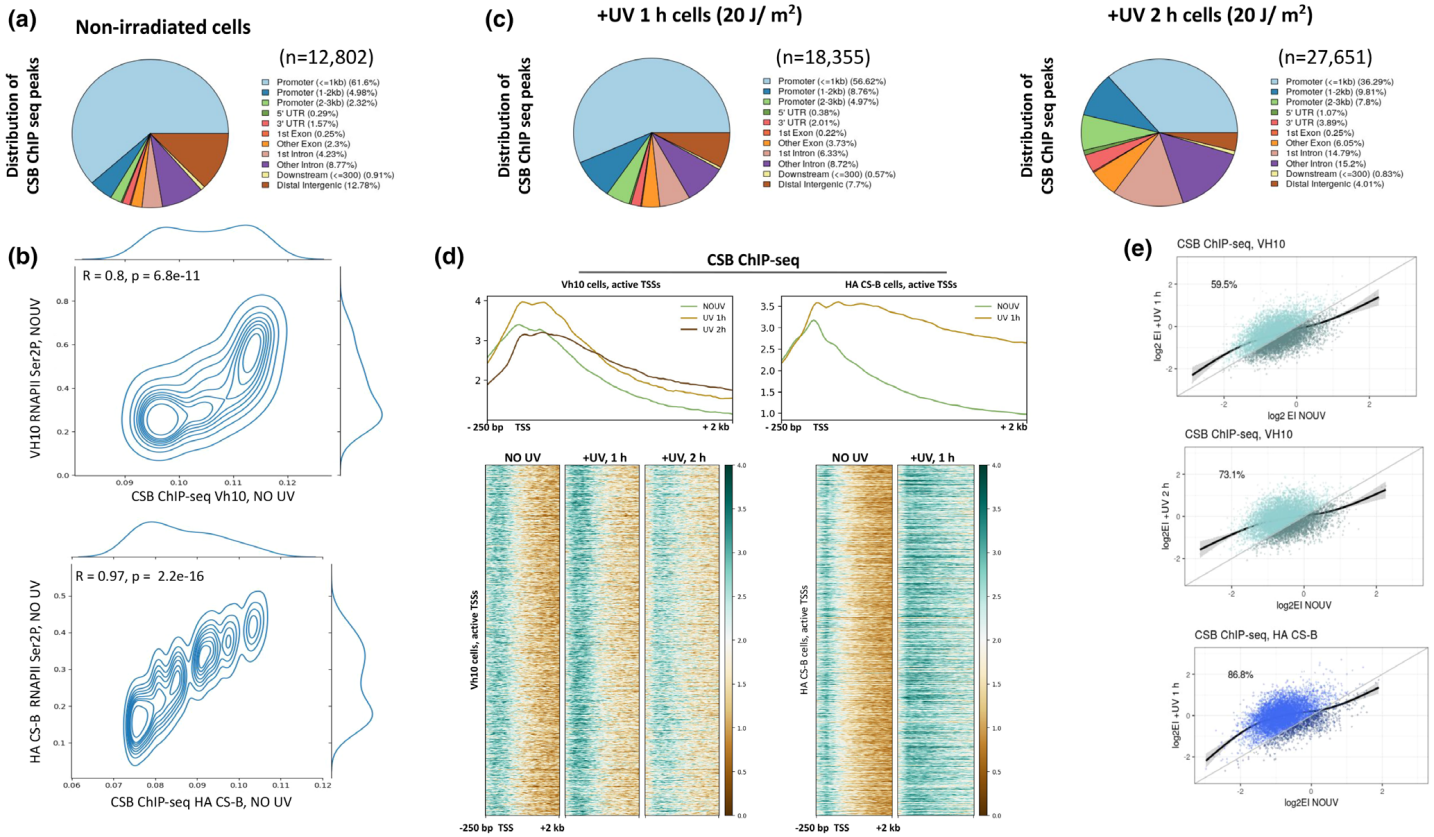
Genome‐wide occupancy of CSB protein in steady‐state and upon UV treatment. (a) Pie chart depicting the distribution of CSB ChIP‐seq peaks (*n* = 12,802) of non‐irradiated normal skin fibroblasts (VH10 htert cells) across distinct genomic categories. (b) Correlation between non‐irradiated RNAPII Ser2P ChIP‐seq and CS‐B ChIP‐seq signal on the EI region. The EI region (−250 bp to 2 Kb) was cut into 50 bp windowed bins and signal of both experiments was counted for VH10 (upper) and HA (lower) data. The resulting vectors were tested for their Pearson correlation. R and p‐values are indicated for each comparison. (c) Pie charts depicting the distribution of CSB ChIP‐seq peaks in irradiated normal skin fibroblasts (Vh10 htert cells) 1 h (*n* = 18,355) and 2 h (*n* = 27,651) after exposure to UV. (d) Average profiles (upper) and heatmap (lower) of the CSB ChIP‐seq in genomic regions from −250 bp to +2 kb around TSSs for the VH10 htert cells (left) and the HA‐CSB cells (right). For irradiated cells, UV dose was 20 J/m^2^. (e) Escape Index (EI) analysis for CSB ChIP‐seq performed in VH10 htert cells (light blue, 1 h vs. NOUV (upper) and 2 h vs. NOUV (middle)) and the HA‐CSB cells (blue, 1 h vs. NOUV (lower)).

Our data so far highlight key differences at the molecular level between normal and CS‐B fibroblasts, in steady state and in response to UV irradiation. The absence of CSB protein is considered the causal factor driving these phenomena, as beyond its function in the TC‐NER cascade, this protein is implicated in RNAPII‐mediated transcription (Batenburg et al., 2021; Boetefuer et al., 2018; Lake et al., 2014; Newman et al., 2006; Selby & Sancar, 1997; Wang et al., 2014; Xu et al., 2020). However, a detailed analysis of the exact role of CSB in steady state and upon UV remains to be elusive. For this reason, we aimed to study and characterize in a precise and genome‐wide manner the distribution of CSB protein in steady state and upon exposure to UV by performing ChIP‐seq experiments in non‐irradiated and irradiated repair‐proficient fibroblast cell lines (Figure S11a). Peak calling analysis (see Methods for details, Table S7) in non‐irradiated fibroblasts revealed that CSB predominantly occupies promoter‐proximal genomic regions (Figure 7a) and that its genomic distribution is similar to that of RNAPII in all cell lines tested (VH10, Pearson correlation coefficient (R): 0.8 and HA CS‐B cells, (R): 0.97 (Figure 7b; Figure S11b). Notably, following UV exposure, we detected an increased number of CSB ChIP‐seq peaks at 1 h and 2 h time points compared to non‐irradiated conditions and a remarkable increase in the relative occupancy of CSB downstream of promoter regions (Figure 7c). Interestingly, we noted that similar to RNAPII Ser2P, the binding profile of CSB was shifted into the gene body after UV, in all cell lines tested (Figure 7d). This was also corroborated by a concomitant increase of EI for CSB ChIP‐seq (Figure 7e), calculated similarly to the EI of RNAPII Ser2P (Figure S1b, see also Methods). Notably, comparing post‐UV EI values from RNAPII Ser2P (data from Lavigne et al., 2017 and here) and CSB ChIP‐seq data, revealed a high correlation (EI 1 h RNAPII Ser2P Vs EI 1 h CSB in VH10 cells R: 0.74, and R: 0.99 for HA CS‐B cells) (Figure S11c). Together, our findings highlight the genome‐wide colocalization between CSB and RNAPII, which seems to increase upon exposure to UV irradiation.

## 4 DISCUSSION

In this report, we provide a comprehensive study of the cellular response to UV in CS‐B fibroblasts and we present evidence implicating CSB protein in transcription‐associated chromatin remodeling at steady state and upon exposure to genotoxic stress. Our data demonstrate that activation of TC‐NER assembly at sites of UV damage‐arrested RNAPII is not a prerequisite for the global transcriptional response and the associated chromatin dynamics observed rapidly upon exposure to genotoxic insult. In contrast to TC‐NER‐proficient normal cells, this response leads to post‐UV prolonged retainment of incomplete nRNAs and elongating RNAPII at the 5′ of active genes.

Our results show that shortly after exposure to UV (30 min–1 h) CS‐B fibroblasts trigger the release of RNAPII from PPP sites to productive elongation (Figure 1). Although in principle similar to the respective response in normal cells (Borisova et al., 2018; Bugai et al., 2019; Lavigne et al., 2017; Liakos et al., 2020), this mechanism was found to involve fewer genes (63%–70% instead of 91%) and occurred in less magnitude (as expressed by EI metrics) in CS‐B fibroblasts (Figure 1d). In line with the enhanced release from PPP sites, it was shown that increased nRNA synthesis occurs in the 5′ ends of genes of both normal and TC‐NER deficient cells, after exposure to UV (Andrade‐Lima et al., 2015; Magnuson et al., 2015).

The aforementioned UV‐triggered de novo influx of RNAPII in gene bodies in CS‐B fibroblasts is accompanied by a slower, compared to normal equivalents, transcription progression, probably due to the inability of CS‐B cells to perform TC‐NER (Figure 2e,f). The marginal progression of elongating RNAPII in the transcribed strand of active genes in CS‐B cells can be attributed, at least in part, to the GG‐NER contribution in the repair of the respective lesions as shown previously by the CPD XR‐seq in normal and CS‐B human fibroblasts (Hu et al., 2015) (Figure S12).

A recent study suggested that the post‐UV phosphorylation and progression of RNAPII into elongation (8 h after exposure) for a subset of genes, occurs in a PAF1C‐dependent manner and through an interaction between CSB and PAF1C which ultimately loads PAF1C at PPP sites (van den Heuvel et al., 2021). The same study showed that in the absence of a functional CSB the presence of RNAPII in intragenic regions of irradiated cells at 8 h post UV was minimal, suggesting that only a limited number of RNAPII molecules can be released into elongation. Even though we cannot exclude such a scenario (especially for the later stage of recovery from UV), our findings demonstrate that, in the early stages of the UV response in CS‐B cells, the release of RNAPII molecules from PPP sites into elongation occurs for a significant number of actively transcribed genes. The fact that in the presence of CSB (TC‐NER proficient background, wild type, and HA CS‐B cells) the vast majority (90%) of active genes show the aforementioned release of RNAPII in response to UV, argues also for a role of CSB in facilitating PPP release early in the response to UV.

The fate of the transcripts that are generated upon the UV‐triggered release of RNAPII into elongation remained for years elusive. We aimed to examine aspects of this problem by conducting sets of Pulse Chase experiments in UV‐treated normal and CS‐B fibroblasts. Interestingly, our results uncovered the retention of nascent transcripts for longer periods in irradiated CS‐B cells compared to normal counterparts (Figure 3b–d). These results indicate that post‐UV synthesized nRNA molecules remain bound to the damage encountering RNAPII or the ones that piled up behind it, or their increased detection reflects a failure of the RNA decay system in the irradiated CS‐B fibroblasts. Interestingly, a recent study showed that upon exposure to UV, an RNA/DNA nuclease, namely EXD2, translocates from mitochondria to the nucleus, interacts with chromatin RNAPII and degrades nRNA (Sandoz et al., 2023). In accordance with our findings, the same study demonstrated that UV‐treated CS‐B cells exhibit a prolonged detection of nascent mRNAs. Whether CS‐B cells are characterized by an aberrant EXD2 activity/targeting upon UV, remains to be elucidated. Similarly, we found that elongating RNAPII molecules exhibited increased retention on the DNA template in irradiated CS‐B fibroblasts compared to normal equivalents, in conditions where further influx of new RNAPII molecules into elongation was blocked (inhibition of PPP release through DRB) (Figure 3e; Figure S4a–d). The inability of CS‐B cells to process and remove the lesion‐stalled RNAPII from the damage site seem to be causally linked to the above observation. It has been shown that in normal UV‐exposed fibroblasts the damage‐encountering RNAPII molecule per se is rapidly ubiquitylated and removed from the lesion site (Chiou et al., 2018; Heilbrun et al., 2020; Nakazawa et al., 2020; Tufegdžić Vidaković et al., 2020). On the contrary, previous studies have reported that this UV‐induced ubiquitylation of the large subunit of RNAPII, which is dependent on CSA protein (Liebelt et al., 2020), is diminished in CS fibroblasts (Bregman et al., 1996; Nakazawa et al., 2020, 2012). Notably, and in line with the above, another recent study (Hansen et al., 2023) demonstrated that both the processing and the degradation of the damage‐arrested RNAPII are greatly affected in cells lacking either CSA or CSB proteins leading to increased stalling of chromatin‐bound RNAPII and shielding of the UV‐induced lesions. In addition to this, the prolonged stalling of the foremost transcribing RNAPII at a damage site in CS‐B cells, is likely to generate structures as R‐loops, which could result in pausing or stalling of the following RNAPII molecules (Belotserkovskii et al., 2018). Since CSB protein has been suggested to be a sensor of R‐loops and a regulator of their repair (Tan et al., 2021; Teng et al., 2018), its absence may result in unresolved R‐loop structures throughout the transcribing genome.

Our findings indicate that the newly synthesized RNA at the promoter‐proximal regions of genes in CS‐B cells derives from a fast shift of promoter‐located RNAPII molecules from the phase of initiation and pausing to that of productive elongation. Indeed, inhibition of PPP release with DRB “rescued” the drop of RNAPII‐hypo, which is observed early after UV (Figure 4). These results suggest that de novo recruitment of RNAPII at promoters of active genes takes place during the early stage of UV response and thus, the process of transcription initiation is not inhibited in irradiated CS‐B cells as previously proposed (Proietti‐De‐Santis et al., 2006; Rockx et al., 2000). Our results are in line with the findings of Andrade Lima et al. (Andrade‐Lima et al., 2015) showing that significant levels of nascent transcription are detectable at the 5′ end of genes in UV‐exposed CS‐B fibroblasts, suggesting that productive transcription elongation rather than initiation are compromised in irradiated CS‐B cells. In support of these findings, an assessment of chromatin accessibility and H3K27ac revealed that chromatin structure at regulatory regions maintains its activity potential after damage induction in CS‐B fibroblasts at the early stages of UV response (Figure 5). On the other hand, UV‐treated normal fibroblasts have been shown to exhibit a more prominent increase in chromatin accessibility at regulatory regions (Liakos et al., 2020; Liu et al., 2021) compared to that observed in irradiated CS‐B cells. We speculate that the stronger chromatin opening at promoters after UV damage induction favors the observed enhanced pause‐release and PIC assembly dynamics in normal cells (Lavigne et al., 2017; Liakos et al., 2020, 2023), resulting in increased fuzziness of +1 nucleosomes (Figure 6). Collectively, these results suggest that although several of the early transcriptional/chromatin characteristics of the UV response seem common between normal and CS‐B fibroblasts, the globality of these responses is compromised in CS‐B cells, revealing a TC‐NER independent role of CSB in shaping the cellular response to UV‐induced genotoxic stress.

Notably, the absence of a functional CSB protein results in more firmly positioned +1 nucleosomes and compact chromatin structure also in steady state. Given the increased association of CSB and RNAPII at genomic regions close to TSS, both in non‐irradiated and UV‐exposed conditions (Figure 7), our data suggest that this interaction may be crucial for the remodeling of nucleosomes at the respective loci in line with an earlier reported study regarding Rad26, the yeast ortholog of CSB (Xu et al., 2020). In support of this, RNAPII progression in transcribed genes was found to be slower in CS‐B fibroblasts compared to normal in unchallenged conditions (Figure 2b,c), suggesting a broader role of CSB protein in facilitating transcription elongation.

## AUTHOR CONTRIBUTIONS

M.F. conceived and led the study and together with M.D.L., K.Z.N.Z., and A.L. designed the experiments and was responsible for the interpretation of the results. A.L., K.Z.N.Z., performed the experiments and analysed the data. Z.S. performed the ChIP‐seq experiments in the HA CS‐B cells. I.T., D.K.K. and G.N. performed part of the western blot and ChIP‐qPCR analyses. N.A. and D.K. performed the statistical and bioinformatics analyses. A.C.S performed part of the bioinformatics analysis. A.L. wrote the manuscript with significant contributions of M.F., N.A., M.D.L. and G.N. All authors discussed the results, reviewed, commented on and approved the final version of the manuscript. M.F. obtained financial support.

## FUNDING INFORMATION

This work was funded by an ERC grant to M.F., Agreement‐309612 (TransArrest), <Matching Funds> to MF funded by National sources, and H.F.R.I. “Research Projects to support Faculty Members and Researchers” Project No: 3199 to MF.

## CONFLICT OF INTEREST STATEMENT

None to declare.

## Supporting information


Figure S1.
Figure S2.
Figure S3.
Figure S4.
Figure S5.
Figure S6.
Figure S7.
Figure S8.
Figure S9.
Figure S10.
Figure S11.
Figure S12.

Table S1.

Table S2.
Table S3.
Table S4.

Table S5.

Table S6.

Table S7.

## Data Availability

The sequencing data have been deposited to the BioSample database with accession ID PRJNA1061543. An overview of the data is available in the following link: https://dataview.ncbi.nlm.nih.gov/object/PRJNA1061543?reviewer=7kr054pud8gveld6k4uc66v2vm The code used is available upon request. Τracks of processed data are available in the following genome browser link: UCSC session.
