# Supplementary material for: Cockayne syndrome B protein is implicated in transcription and associated chromatin dynamics in homeostatic and genotoxic conditions
Source: Aging Cell. 2024 Oct 6;24(1):e14341. doi: 10.1111/acel.14341 (PMC11874911; doi:10.1111/acel.14341)
Supplement: Supplementary file 1 — Figure S1. Figure S2. Figure S3. Figure S4. Figure S5. Figure S6. Figure S7. Figure S8. Figure S9. Figure S10. Figure S11. Figure S12. [file ACEL-24-e14341-s005.pdf]

Supplementary Figure 1

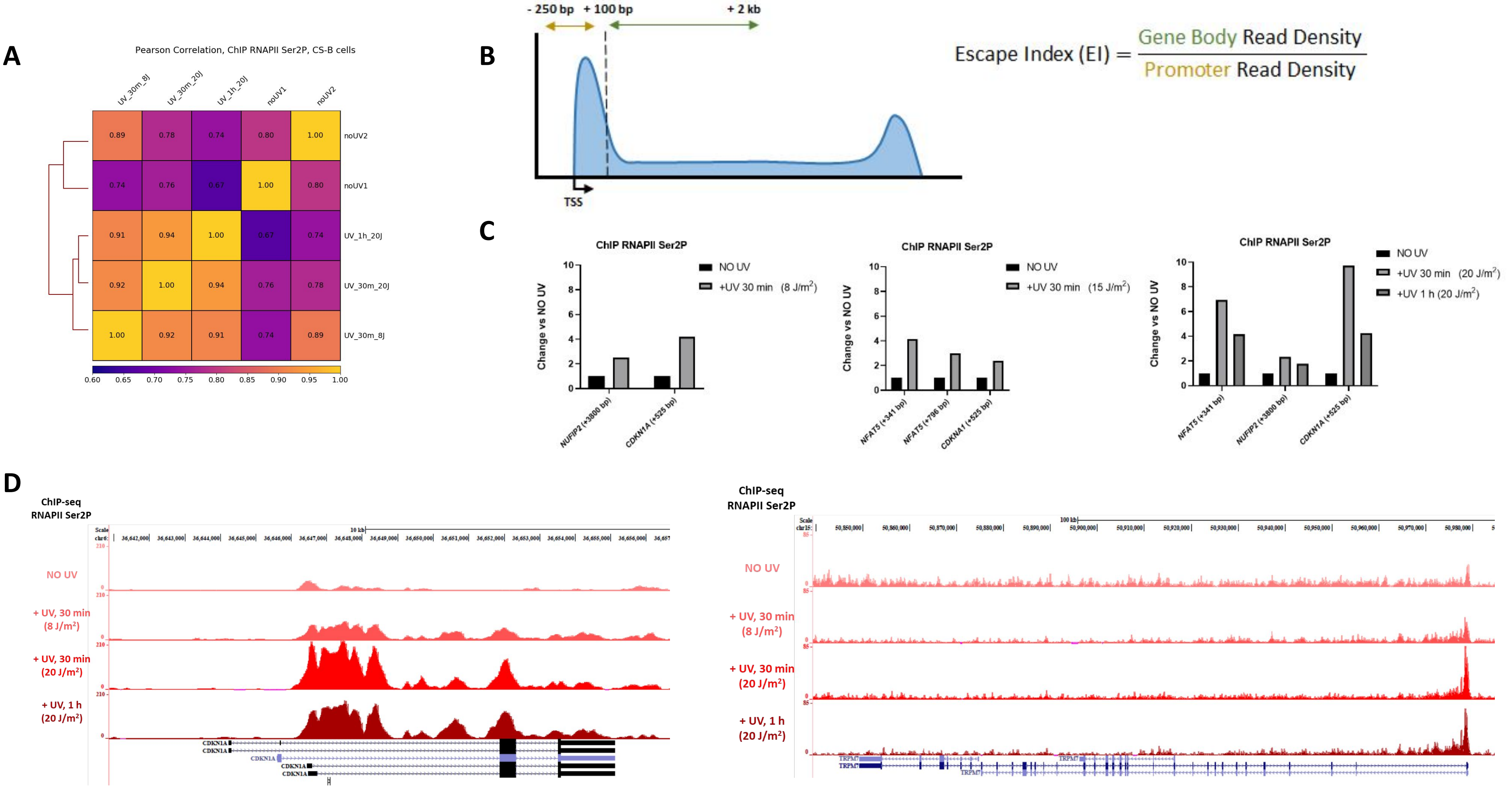

Supplementary Figure 2

A

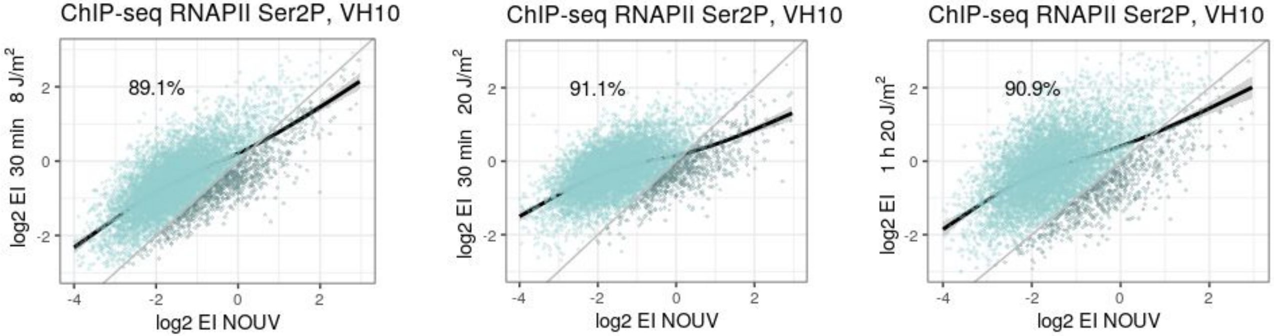

B

RNAPII Ser2P ChIP-seq, HA CSB
Pearson Correlation

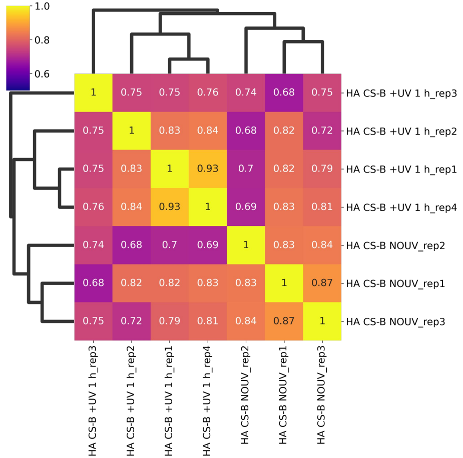

Supplementary Figure 3

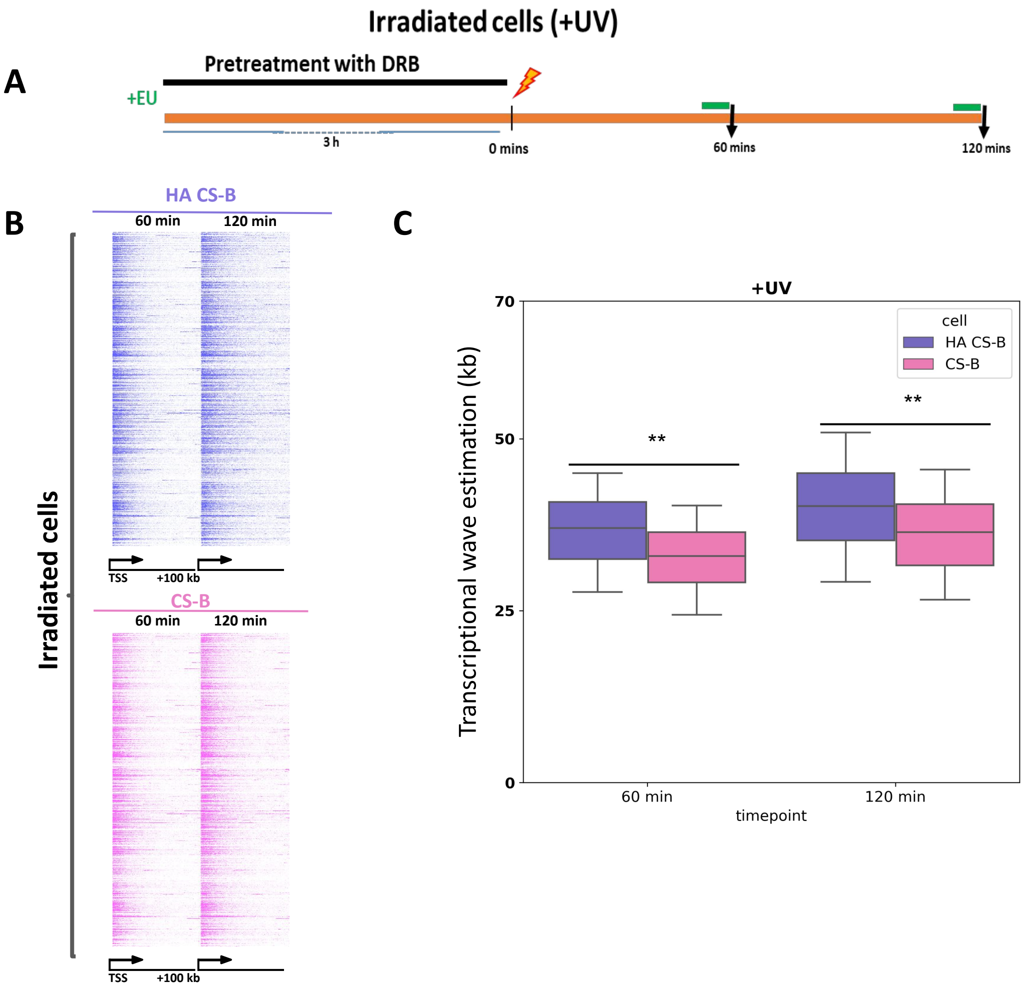

Supplementary Figure 4

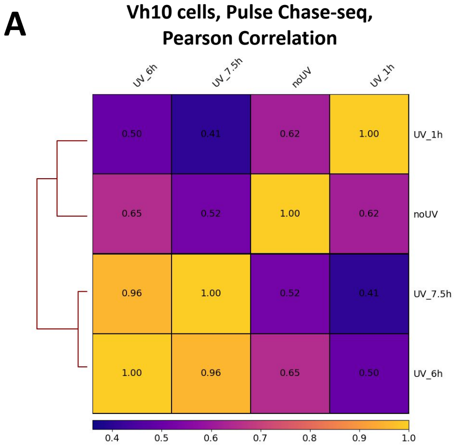

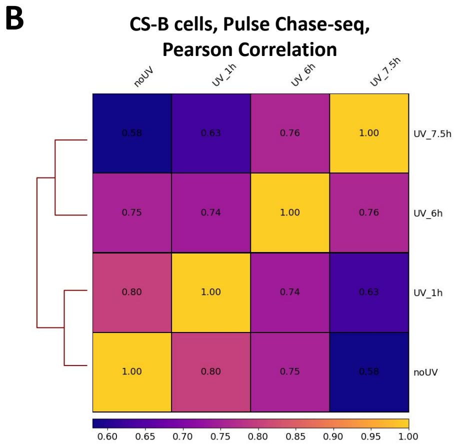

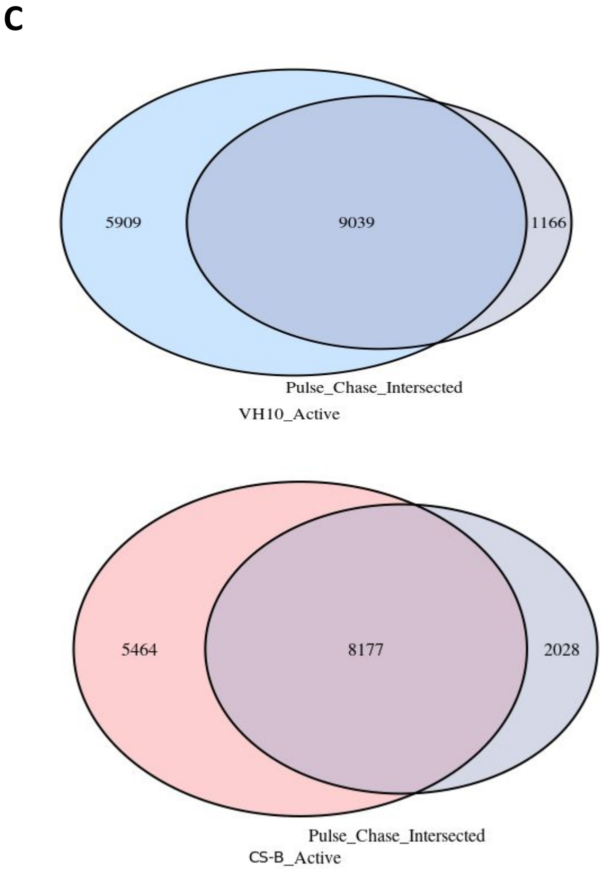

Supplementary Figure 5

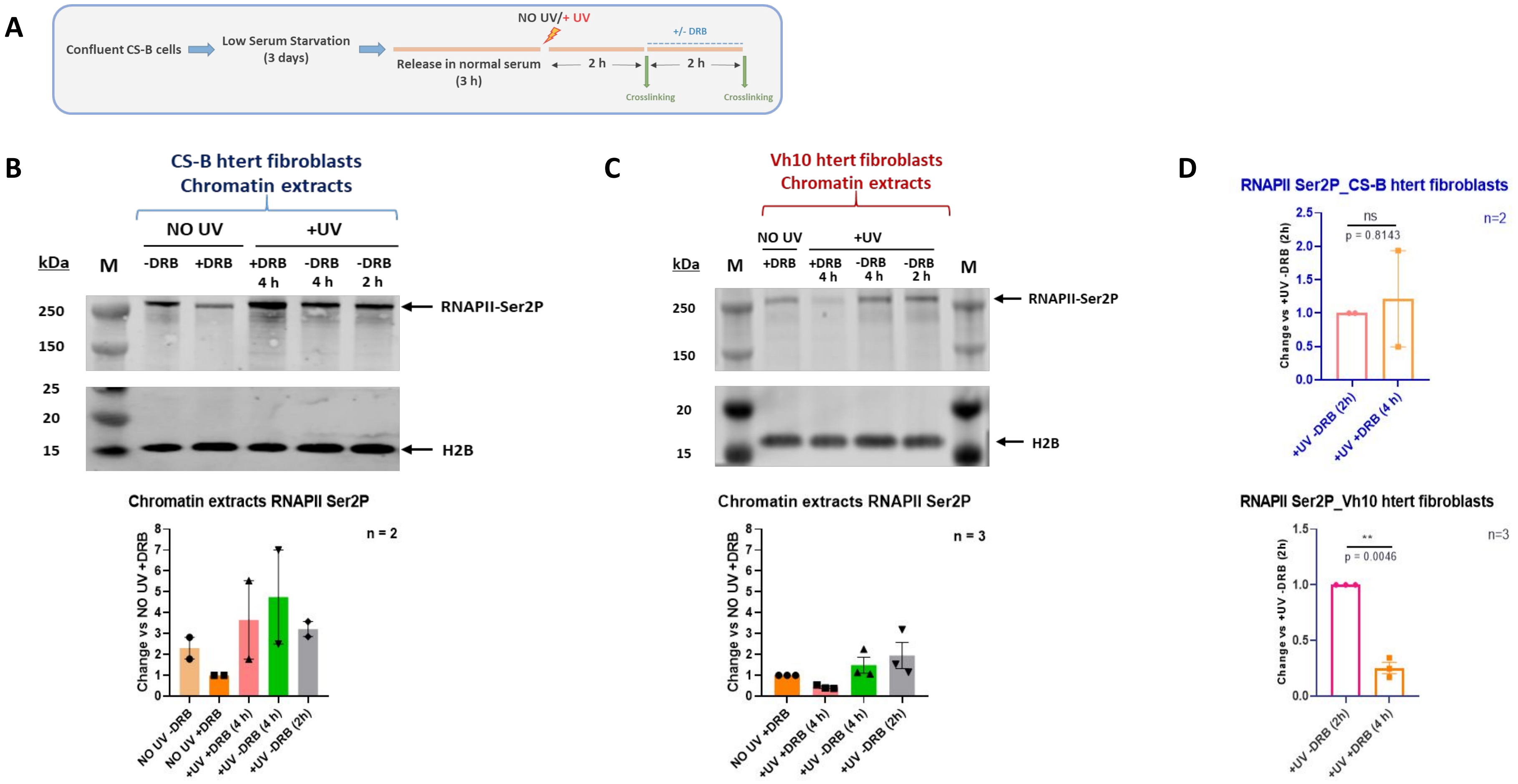

Supplementary Figure 6

A

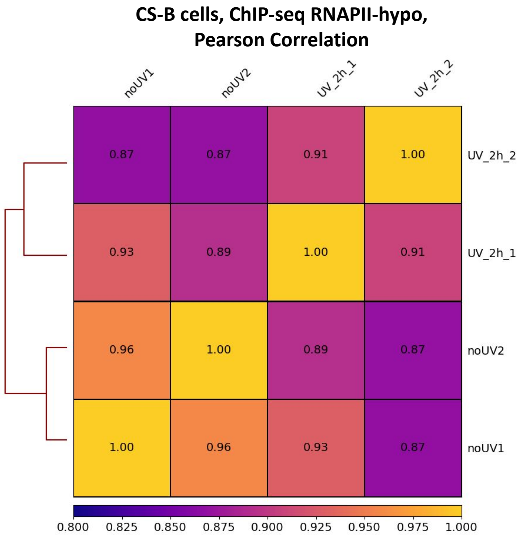

Supplementary Figure 7

A

CS-B cells, ATAC-seq, Pearson Correlation

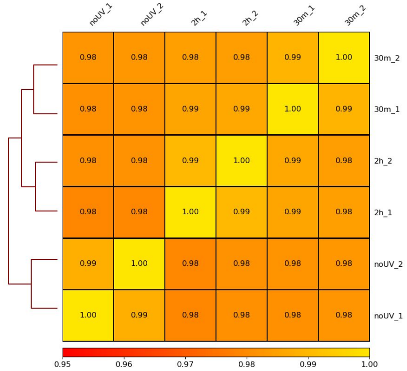

B

Fragment Distribution, ATAC-seq
CS-B cells, NO UV

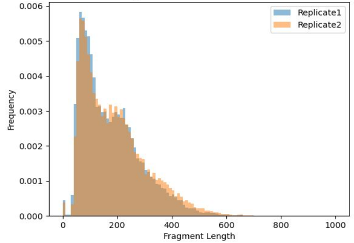

Fragment Distribution, ATAC-seq
CS-B cells, + UV, 30 min

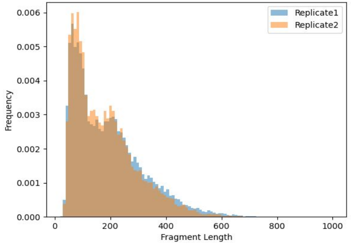

Fragment Distribution, ATAC-seq
CS-B cells, +UV, 2 h

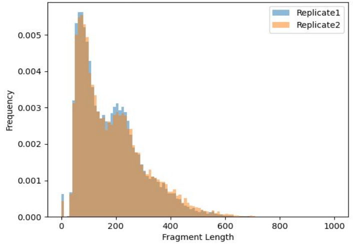

C

NO UV ATAC-seq peaks

n=174,098

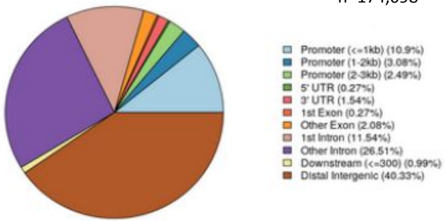

+UV 30 min ATAC-seq peaks

n=176,755

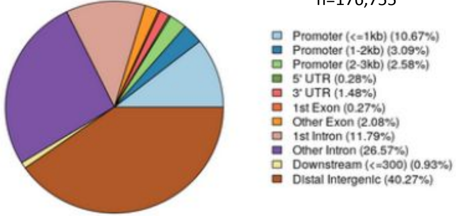

+UV 2h ATAC-seq peaks

n=187,947

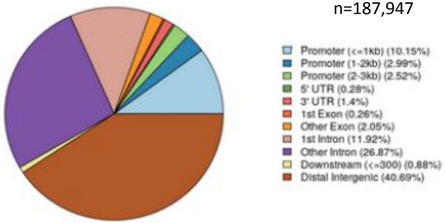

Supplementary Figure 8_ REVISED

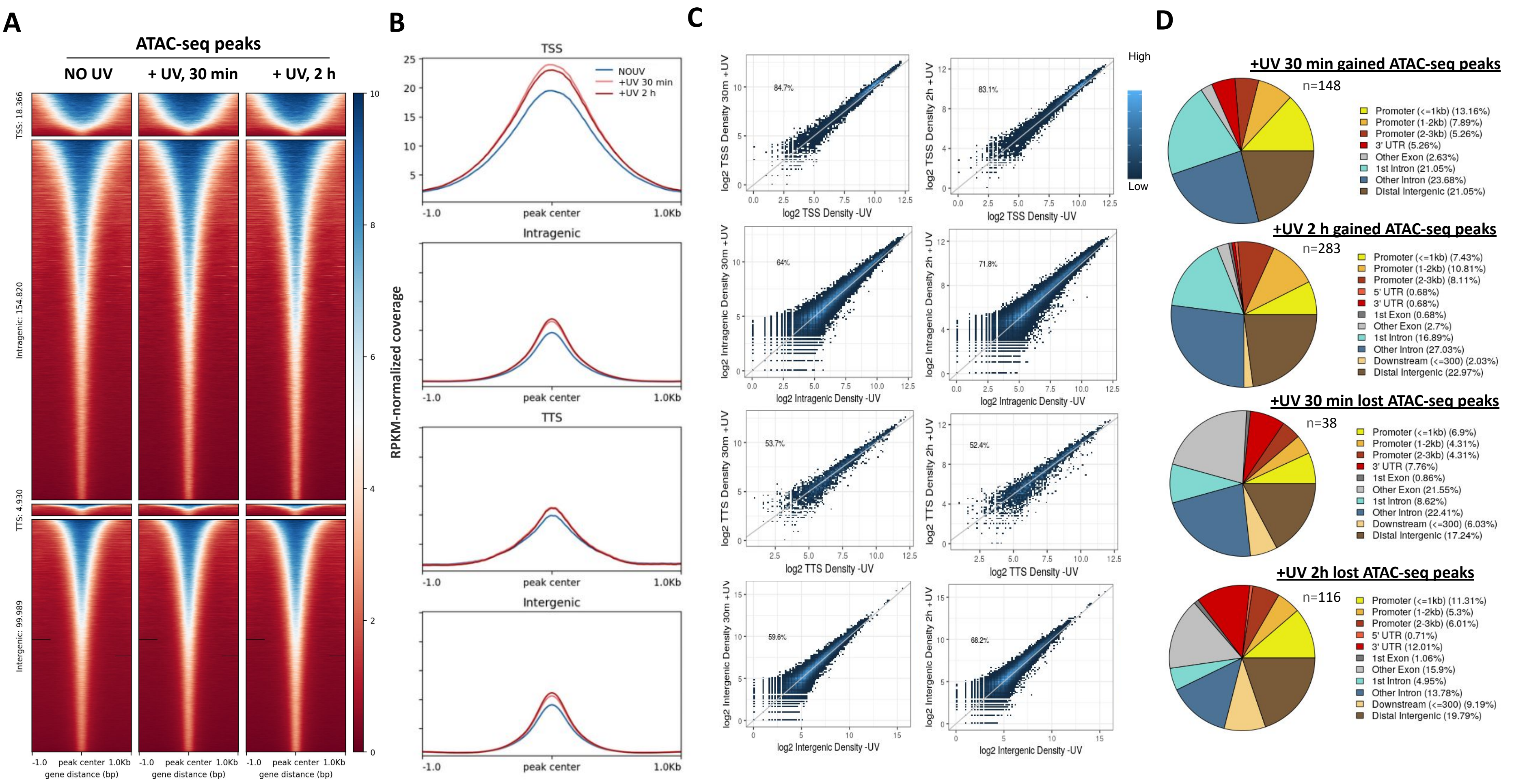

Supplementary Figure 9

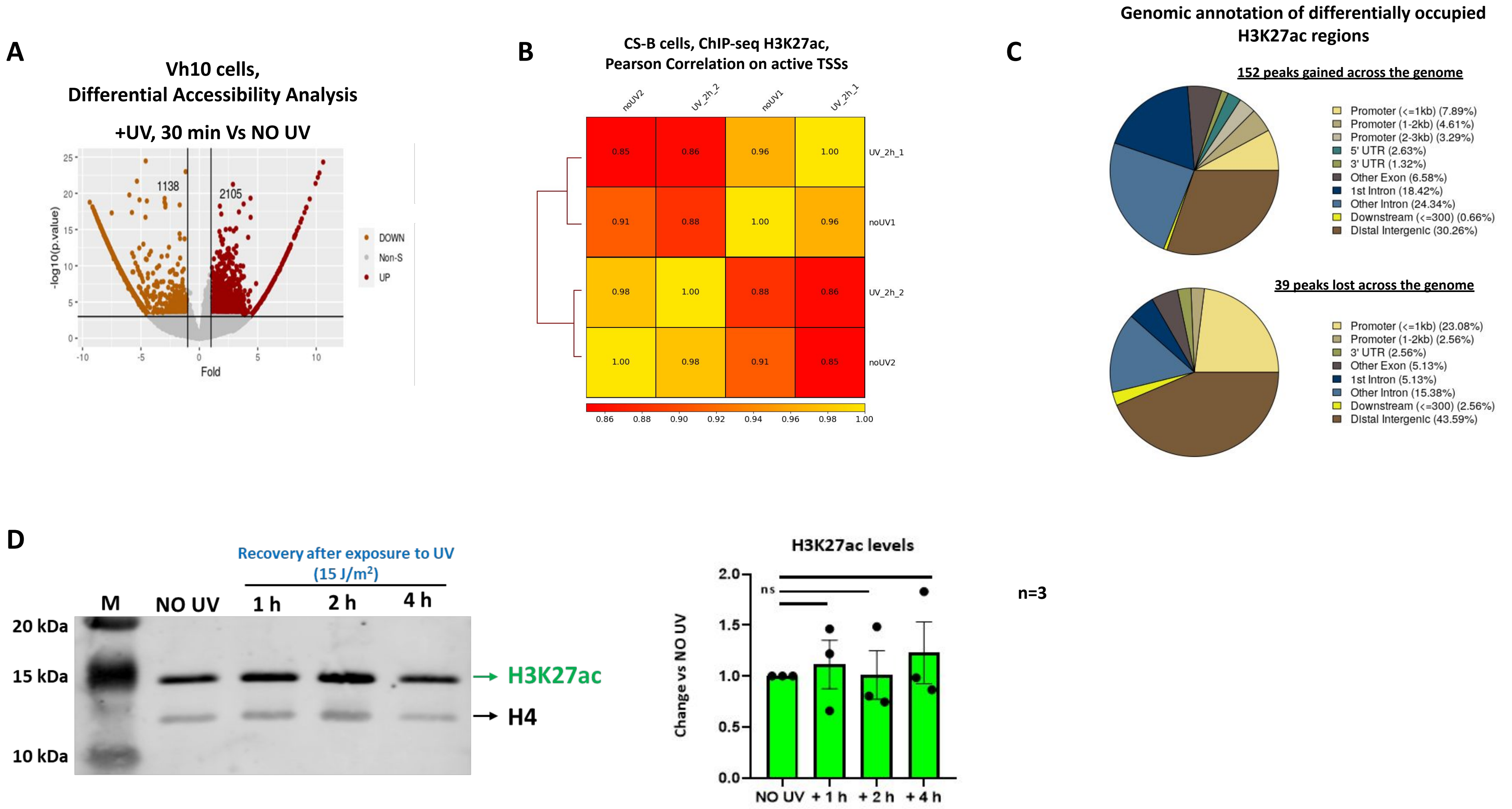

Supplementary Figure 10

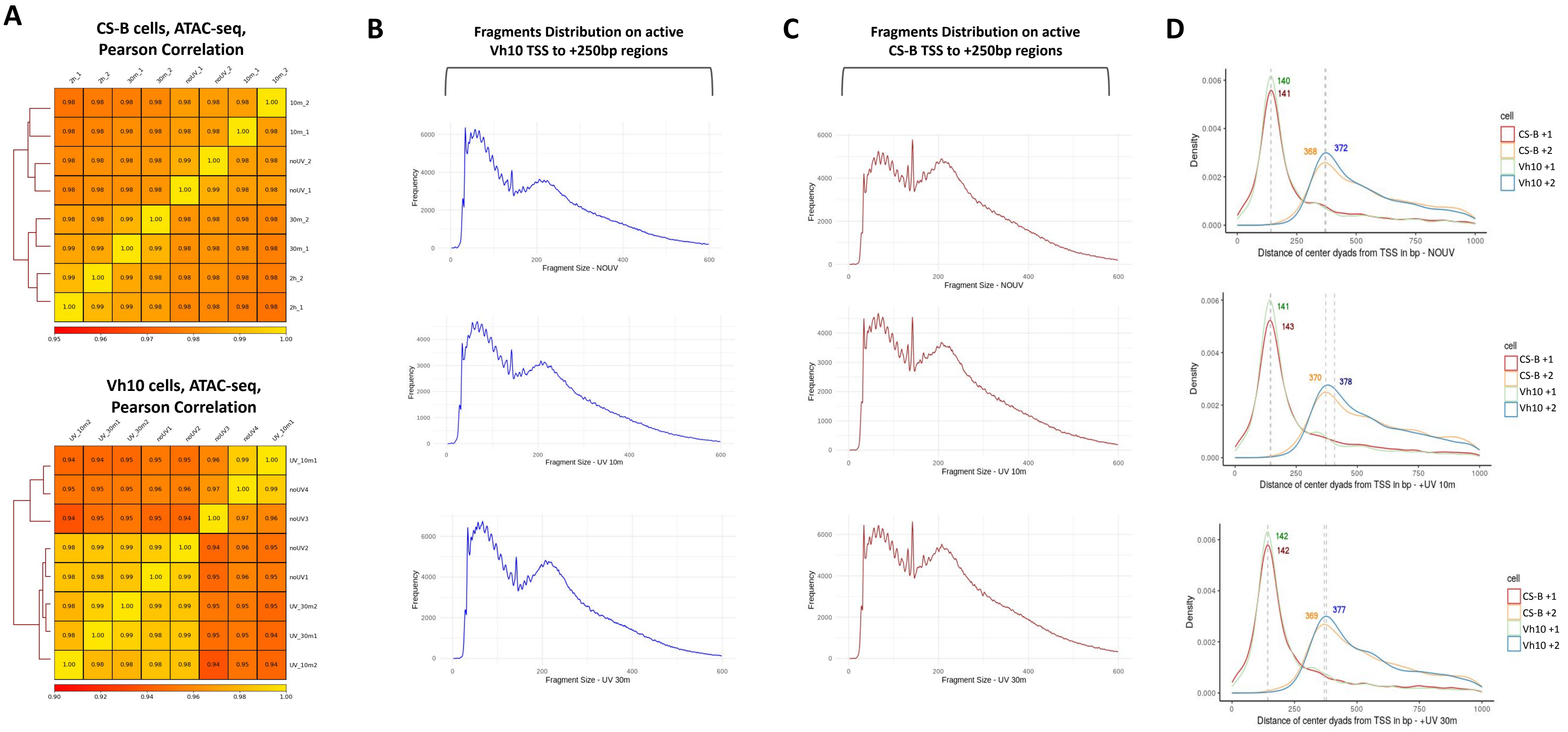

Supplementary Figure 11

A

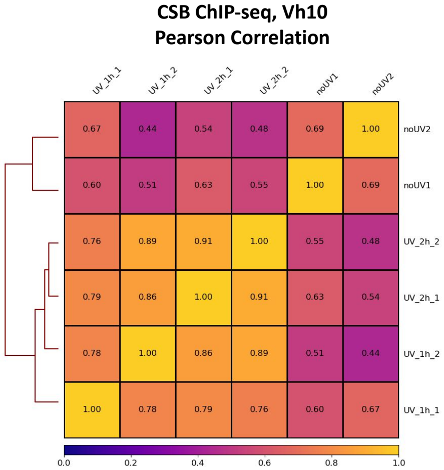

B

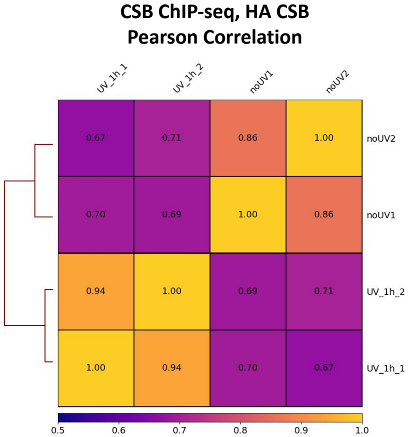

C

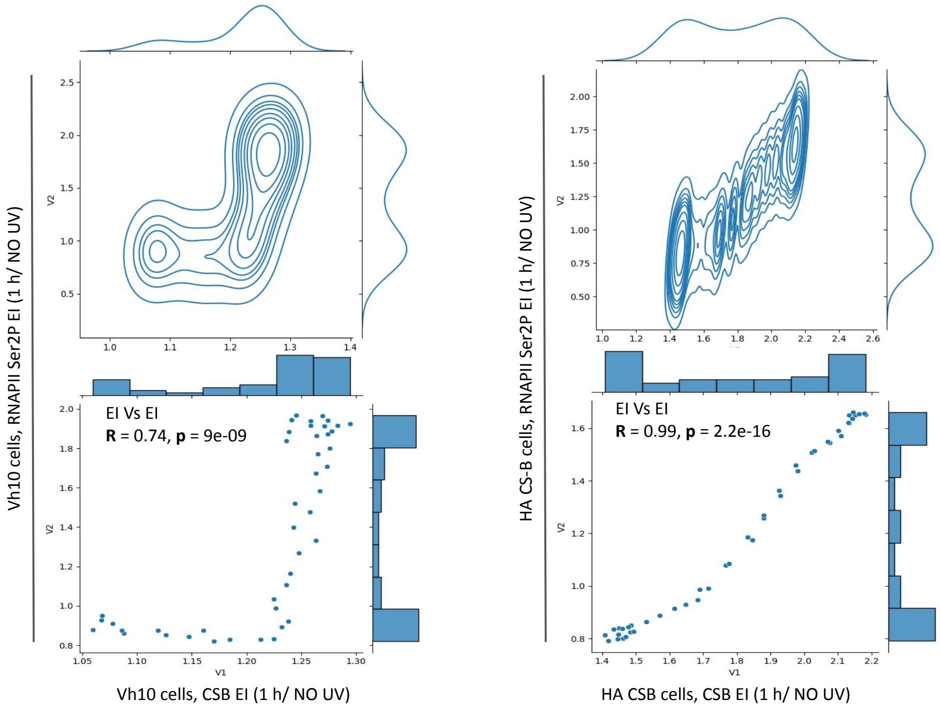

Supplementary Figure 12

A

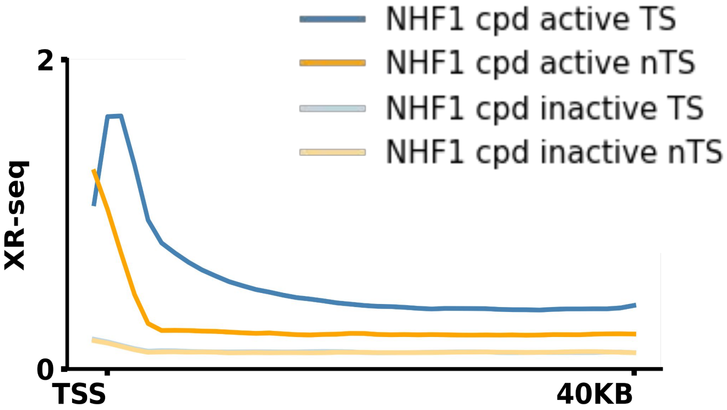

B

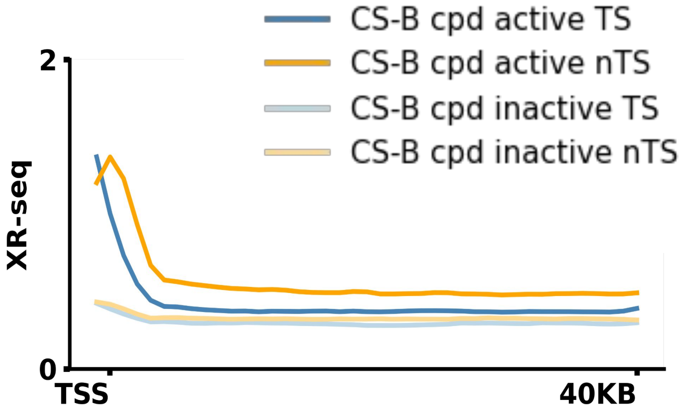

Supplementary Figure Legends

Supplementary Figure 1:

A. Correlation plot of RNAPII-ser2 ChIP-seq experiments in CS-B fibroblasts at genomic regions -250 bp to +2 kb around active TSSs.

B. Escape Index (EI) calculation. The EI is calculated from the ratio of the density of reads in the gene body (from 100 bp to 2 kb downstream of the TSS), over the density of reads at the promoter proximal region (from 250 bp upstream to 100 bp downstream of the TSS).

C. ChIP-qPCR analysis of RNAPII Ser2P enrichment in specific genomic regions of CS-B htert fibroblasts before (NO UV) and after treatment with 8 J/m^2 (Left), 15 J/m^2 (Middle), and 20 J/m^2 (Right) UV. Distance of each primer set from the respective gene TSS is indicated. Fold Changes were normalized to a negative primer (ChIA neg) and expressed as compared to control (NO UV) for each different UV dose.

D. UCSC snapshots depicting RNAPII Ser2P ChIP-seq signal in non-irradiated (NO UV) and irradiated (+UV) CS-B fibroblasts for indicative genes. UV dose and recovery time are depicted. For *CDKN1A* (left) the genomic region amplified in ChIP-qPCR reactions is illustrated below the schematic.

Supplementary Figure 2:

A. Escape Index (EI) analysis for RNAPII Ser2P ChIP-seq between irradiated ((left) +UV 8 J/m^2, 30 mins), ((middle) +UV 20 J/m^2, 30 mins), ((right) +UV 20 J/m^2, 1 h) and non-irradiated (NO UV) Vh10 htert cells. Percentages of loci with increased EI for each comparison are shown in lighter color. Data were obtained from Lavigne et al., 2017.

B. Pearson coefficient correlation plot of the RNAPII Ser2P ChIP-seq biological replicates for non-irradiated (NO UV) and irradiated (+UV, 1 h, 20 J/m^2) HA-CS-B fibroblasts.

Supplementary Figure 3:

A. Experimental timeline depicting DRB/GRO-seq experimental setup in irradiated HA CS-B SV40 (wild type) and CS-B SV40 skin fibroblasts. UV dose was 12 J/m^2. EU labeling periods are indicated in green.

B. Heatmaps illustrating GRO-seq signal and transcription wave progression at the indicated time points for irradiated (12 J/m^2) HA CS-B (upper) and CS-B SV40 (lower) skin fibroblasts. Depicted genomic regions are indicated.

C. Progression of transcriptional waves based on Hidden Markov Model (HMM) for the two cell lines, in the indicated time points for non-irradiated HA CS-B SV40 and CS-B SV40 skin fibroblasts. Asterisks (*) show the paired t-test significance of the difference between the two cell lines for the time points indicated.

Supplementary Figure 4:

A. Correlation plot of Pulse Chase-seq experiments performed in Vh10 htert fibroblasts, at genomic regions -250 bp to +2 kb around TSSs.

B. Same as in A, but for CS-B htert skin fibroblasts.

C. Intersection of the active VH10 (blue) and CS-B (pink) gene lists and the genes used in the Pulse-Chase (grey) analysis (see Methods).

Supplementary Figure 5:

A. Experimental timeline. For irradiated cells UV dose was 15 J/m^2.

B. (Upper) Western Blot analysis of chromatin extracts of CS-B fibroblasts for elongating RNAPII (RNAPII-Ser2P) treated as explained in experimental timeline. H2B was used as loading control. (Lower) Quantification of RNAPII-Ser2P levels for Western Blot depicted in the upper panel. Results

are expressed as compared to non-treated sample (NO UV, -DRB). Error bars represent standard error of the mean (S.E.M). The blot is representative of two independent biological experiments (n=2).

C. As in B but for Vh10 htert fibroblasts (normal). Experimental conditions are indicated. Quantification results are expressed as compared to NO UV +DRB sample. Error bars represent standard error of the mean (S.E.M). The blot is representative of three independent biological experiments (n=3).

D. Quantification of the average signal detected for RNAPII Ser2P in +UV +DRB (4h) condition of (B) and (C) as compared to +UV -DRB (2h) condition, for CS-B htert (Upper) and Vh10 htert (Lower) skin fibroblasts. P-values of two-tailed t-tests are indicated.

Supplementary Figure 6:

A. Correlation plot of the RNAPII-hypo ChIP-seq biological replicates, in non-irradiated (NO UV) and irradiated (+UV, 2 h, 15J/m^2) CS-B htert fibroblasts, at the regions used for EI calculations around TSSs (-250 bp to +2Kb).

Supplementary Figure 7:

A. Correlation plot of the ATAC-seq biological replicates performed in CS-B fibroblasts at genomic regions -250 to +2 kb around TSSs.

B. Fragment size distribution plot for ATAC-seq experiments performed in non-irradiated (NO UV, left) and irradiated (+UV, 30 min (middle) and 2 h (right)) CS-B fibroblasts.

C. Pie charts illustrating the distribution of ATAC-seq peaks across selected genomic regions, for each different experimental condition (NO UV, +UV 30 min, +UV 2 h) in CS-B fibroblasts.

Supplementary Figure 8:

A. ATAC-seq signal in genomic regions 1 kb around peaks detected in each experimental condition (NO UV, +UV 30 min, +UV 2 h) in CS-B fibroblasts. ATAC-seq peaks were grouped to TSS, Intergenic, TTS or Intragenic according to their genomic localization. Numbers of peaks for each category are indicated.

B. Average profiles of ATAC-seq signal, in genomic regions depicted in Supplementary Figure 8A.

C. Heat-density scatter plots of the +UV/-UV signal ratio at all groups of Supplementary Figure 8A.

D. Pie charts depicting annotation of significantly gained or lost ATAC-seq peaks after UV-irradiation in CS-B fibroblasts, according to the Differential Accessibility analysis of Figure 5D. “Downstream” refers to genomic regions downstream of gene end. Numbers of differential accessible loci for each category are indicated.

Supplementary Figure 9:

A. Volcano plot depicting the differential accessibility analysis for irradiated (+UV, 30min) and control (non-irradiated, NO UV) normal (Vh10) fibroblasts. Genomic regions showing significantly (p-value threshold = 0.001, FC = 1) increased (dark red) or decreased (brown) chromatin accessibility, are depicted, and their numbers are indicated.

B. Correlation plot of H3K27ac ChIP-seq biological replicates performed in CS-B fibroblasts in genomic regions the regions -250 bp to +2 kb around TSSs.

C. Pie charts depicting the annotation of the distribution of CS-B H3K27ac ChIP-seq peaks gained (152) or lost (39) after irradiation according to the Differential binding analysis in Figure 2 (F).

D. (Left) Western Blot analysis of histone extracts, showing bulk H3K27ac levels in non-irradiated (NO UV) and irradiated CS-B fibroblasts. Histone 4 (H4) was used as loading control. (M) Protein marker. The figure is representative of three independent biological experiments. (Right) Quantification of bulk H3K27ac signal in CS-B fibroblasts, expressed as compared to non-irradiated cells (NO UV). Error bars represent standard error of the mean (S.E.M.)

Supplementary Figure 10:

A. Correlation plot of the ATAC-seq biological replicates performed in CS-B htert (Upper) and normal (Vh10 htert) fibroblasts (Lower) and at genomic regions -250 bp to +2 kb around TSSs (bin size = 500 bp).

B. Fragment size distribution plot for ATAC-seq experiments performed in non-irradiated (NO UV, Upper) and irradiated (+UV, 10 min (middle) and 30 min (lower)) CS-B htert fibroblasts. Genomic regions from TSS to +250 bp are depicted.

C. Same as in B but for normal (Vh10 htert) fibroblasts.

D. Distances between the active non-overlapping TSSs of genes(see also Methods) and their +1 and +2 dyad centers called by NucleoATAC, in base pairs (bp), in each cell line (CS-B, Vh10) for non-irradiated (NO UV, Upper) and irradiated (+UV, 10 min (middle) and 30 min (lower)) conditions. Maximum density point (y-axis) represents the most common distance found.

Supplementary Figure 11:

A. Correlation plot of the CSB ChIP-seq replicates performed in Vh10 htert cells at genomic regions -250 bp to +2 kb around active TSSs.

B. Same as in A but for CSB ChIP-seq experiments performed in HA CSB fibroblasts.

C. Pearson correlation between the EI (1h/NOUV) of the RNAPII-Ser2P ChIP-seq and CSB ChIP-seq in VH10 htert (left) and HA CSB (right) cells. The signal counts were measured on 50 bp windowed bins in the aforementioned area and R and p-values of the Pearson tests are indicated for each comparison.

Supplementary Figure 12:

A. Average profiles depicting CPD XR-seq signal (data obtained from Hu et al., 2015) in NHF1 human fibroblasts, for transcribed (TS) and non-transcribed (nTS) strand of active and inactive genes larger than 40 kb.

B. Same as in A, but for CS-B fibroblasts. Data were obtained from Hu et al., 2015.

<u>Supplementary References</u>

Hu, J., Adar, S., Selby, C. P., Lieb, J. D., & Sancar, A. (2015). Genome-wide analysis of human global and transcription-coupled excision repair of UV damage at single-nucleotide resolution. *Genes and Development*. https://doi.org/10.1101/gad.261271.115

Lavigne, M. D., Konstantopoulos, D., Ntakou-Zamplara, K. Z., Liakos, A., & Fousteri, M. (2017). Global unleashing of transcription elongation waves in response to genotoxic stress restricts somatic mutation rate. *Nature Communications*, *8*(1). https://doi.org/10.1038/s41467-017-02145-4
